# Lipoxygenase LOX3 Is the Enigmatic Tocopherol Oxidase in Runner Bean (*Phaseolus coccineus*)

**DOI:** 10.3390/antiox13030301

**Published:** 2024-02-29

**Authors:** Jerzy Kruk, Paweł Jedynak, Sylwia Kędracka-Krok, Renata Szymańska, Michał Gabruk

**Affiliations:** 1 Department of Plant Physiology and Biochemistry, Faculty of Biochemistry, Biophysics and Biotechnology, Jagiellonian University, Gronostajowa 7, 30-387 Kraków, Poland; pawel.jedynak@uj.edu.pl (P.J.); michal.gabruk@uj.edu.pl (M.G.); 2 Department of Physical Biochemistry, Faculty of Biochemistry, Biophysics and Biotechnology, Jagiellonian University, Gronostajowa 7, 30-387 Kraków, Poland; sylwia.kedracka-krok@uj.edu.pl; 3 Faculty of Physics and Applied Computer Science, AGH University of Krakow, Reymonta 19, 30-059 Kraków, Poland; renata.szymanska@fis.agh.edu.pl

**Keywords:** *Phaseolus coccineus*, oxidase, tocopherol, lipoxygenase, mass spectrometry

## Abstract

Purification of extracts from the etiolated seedlings of runner bean (*Phaseolus coccineus*), coupled with mass spectrometry analysis of proteins revealed that the enzyme responsible for tocopherol oxidation activity is lipoxygenase, an enzyme known for enzymatic lipid peroxidation of unsaturated lipids. Biochemical analysis of the activity, along with the expression profile of three *LOX* isoforms (*LOX1*, *LOX2*, *LOX3*) in various parts of the etiolated seedlings, revealed that *LOX3* was the major isoform expressed in the epicotyls, indicating that this isoform was responsible for the tocopherol oxidation activity; in the primary leaves, besides *LOX3*, the other two isoforms might have also contributed to the activity. The experiments performed in the model systems showed that unsaturated lipids were not required for the tocopherol oxidase activity, but that lipids were necessary to provide an optimal, hydrophobic environment of the substrate for the reaction. The experiments on lipoxygenase and tocopherol oxidase activities in the leaves of light-grown *P. coccineus* plants during aging and during storage of the extracts from etiolated seedlings showed that the activity of the first reaction decreased considerably faster than the latter, indicating different mechanisms of both reactions performed by the same enzyme. As LOX3 was shown to occur in the apoplast of the related species *P. vulgaris*, the question as to the physiological function of LOX3 in the tocopherol oxidation activity in *P. coccineus* is discussed.

## 1. Introduction

Tocopherols (Tocs), along with tocotrienols belong to a group of tocochromanols whose main function in plants is antioxidant action, but signalling functions of these compounds have also been suggested [1,2,3]. Although their biosynthesis is relatively well recognized [4,5], the catabolism of these prenyllipids is poorly investigated. The most well-known products of Tocs oxidation are the corresponding tocopherolquinones which are formed mainly during the scavenging reaction of singlet oxygen [6,7], while hydroxy-plastoquinones (PQ-C) are the corresponding products of the reaction of plastoquinol with singlet oxygen [7,8,9]. All these reactions are non-enzymatic. Additionally, there are data indicating a possibility of enzymatic oxidation of Tocs in plants. In the early 1960s, it was demonstrated that cell-free extracts from etiolated shoots or leaves of several plant species (e.g., *Pisum sativum*, *Phaseolus vulgaris*, *Xanthium struminarium*) showed activity in Toc degradation [10,11,12,13,14,15]. The enzyme responsible for this reaction was named tocopherol oxidase [11] as it a catalyzed oxidation of α-Toc to α-tocopherolquinone by molecular oxygen. Besides oxygen, the oxidase was shown to require phospholipids for its activity [16]. The enzyme has been found in all parts of plants, like roots, stems, leaves, flowers and fruits [11,15]. Recently, tocopherol oxidase activity has been found in corn germs [17]. Our previous work in this subject [18] allowed further characterisation of the oxidase, in regard to substrate specificity and products formed; however, purification and identification of the enzyme was unsuccessful due to the instability of the oxidase activity.

In the current research, we found conditions for preserving the enzyme stability which allowed for partial purification and identification of the enzyme by mass spectrometry. The oxidase was further characterized biochemically and physiologically; its possible function is also discussed.

## 2. Materials and Methods

### 2.1. Plant Material and Extract Preparation

The extracts from etiolated seedlings of *Phaseolus coccineus* cv. ‘Piękny Jaś’ were prepared by a modified procedure described earlier [18]. The seeds were provided by W. Legutko Przedsiębiorstwo Hodowlano-Nasienne Sp. z o.o. (Jutrosin, Poland) and were used immediately after they were received. The percent of germination was ca. 80%. The plants were grown for the indicated time at 20 ± 2 °C. The shoots (primary leaves with the upper ± half of the epicotyl) were homogenized for 15 s with a blender (Philips, Warsaw, Poland) or ground in a mortar in 50 mM citrate–phosphate buffer pH 5.5, using 2 mL of the buffer for every 1 g of fresh weight of the tissue [15]. The homogenate was centrifuged on a Beckman J2-MC centrifuge (Beckman Coulter, Inc., Palo Alto, CA, USA) at 30,000× *g* for 40 min at 4 °C and the obtained supernatant was used for the experiments. In some experiments, where indicated, only primary leaves or epicotyls were used for the preparation of the homogenate. When light-grown plants (100 μmol quanta/m^2^/s, 20 ± 2 °C, 12 h photoperiod) of different ages were used for experiments, the oldest leaves were always used first.

### 2.2. Determination of Tocopherol Oxidase and Lipoxygenase Activity

For the determination of tocopherol oxidase activity, liposomes/micelles were formed by mixing of appropriate amounts of tocopherol and lipids in ethanol and slow injection of the mixture into the investigated extract. The lipids used were egg yolk lecithin (EYL) (Sigma-Aldrich, Poznań, Poland, type V-E), dilinoleoyl-phosphatidylcholine (DLPC) (Avanti Polar Lipids, Birmingham, AL, USA), diheptanoyl-phosphatidylcholine (DHPC) (Avanti Polar Lipids, Birmingham, AL, USA), dimyristoyl-phosphatidylcholine (DMPC) and linoleic acid (LA) (Fluka, Buchs, Switzerland). The final volume of the reaction mixture was 0.5 or 1 mL and the injection volume was 12.5 or 25 µL, respectively; the final lipid concentration was 1 mM and that of α-Toc—100 µM. The reaction was performed at 20 ± 2 °C, unless otherwise stated, under magnetic stirring. As we have found that the that oxygen concentration in the homogenate is very low due to the action of lipoxygenase, the homogenate was air saturated before the activity measurements, using an air-pump for 5 min under magnetic stirring.

The progress of the reaction in the investigated samples was followed by measurements of α-Toc and α-tocopherolquinone content with HPLC. The samples for HPLC analysis were taken at the indicated time of the reaction. The samples (100 µL) were mixed with 100 µL of ethanol in Eppendorf tubes, vortexed for 60 s, following the addition of 300 µL of ethyl acetate and subsequently vortexed for 60 s. The mixture was centrifuged (30 s × 10,000× *g*, Eppendorf Mini SpinPlus) and 150 µL of the upper phase was dried under a stream of nitrogen, dissolved in the HPLC solvent (acetonitrile/methanol/water; 72/8/1, *v*/*v*) and analyzed by HPLC. The HPLC measurements were performed using 100 µL loop, Jasco PU-980 pump and UV–VIS detector system UV-970, Shimadzu RF10-AXL fluorescence detector (excitation/emission detection at 290/330 nm), Teknokroma (Barcelona, Spain) C18 reverse-phase column (Nucleosil 100, 250 × 4 mm, 5 µm), isocratic solvent system—acetonitrile/methanol/water (72/8/1, *v*/*v*/*v*) at a flow rate of 1.5 mL/min, as described previously [19].

If not otherwise indicated, the tocopherol oxidase activity was determined in EYL liposomes, using undiluted extracts. Similarly, if not otherwise indicated, lipoxygenase activity was measured using a Clark-type electrode (Hansatech, Norfolk, UK) using 5-fold diluted extract in the citric buffer pH 5.5 and 1 mM LA as a substrate.

### 2.3. Chromatography

The obtained extract was subject to chromatography on DEAE-Sepharose in 50 mM citric buffer (pH 5.5) with the elution of proteins with increasing concentration of arginine (25, 50 and 100 mM) in the same buffer. The eluted fractions were monitored for protein concentration (A_280_, Bradford method, A_595_—turbidity) and tocopherol oxidase activity using a standard test in EYL/Toc liposomes. The chromatography was performed in a cold-room. The fractions with the highest oxidase activity were concentrated on Vivaspin 20 100 kDa MWCO (GE Healthcare, Little Chalfont, Buckinghamshire, UK) and subject to MS analysis.

### 2.4. Protein Identification by Mass Spectrometry

For in-solution digestion of 15 μg protein samples, proteins were initially reduced with dithiothreitol (DTT) in 50 mM ammonium bicarbonate (ABC) (the final concentration of DTT was 10 mM) for 40 min at 56 °C, followed by alkylation with iodoacetamide (IAA) in 50 mM ABC (the final concentration of IAA was 20 mM) for 30 min in the dark at room temperature (RT). Subsequently, the proteins were precipitated with 6 volumes of ice-cold acetone, vortexed and incubated overnight at −20 °C. Following this, the samples were centrifuged at 15,000× *g* for 10 min. The pellet was then resolubilized in 30 μL of 50 mM ABC. Trypsin digestion was performed as described above, with a protein/trypsin ratio of 100:1. Digestion was stopped by adding up to 5% formic acid (FA) and the obtained peptides were dried.

Bands from SDS-PAGE, corresponding in the molecular mass to lipoxygenases (80–100 kDa), were excised and destained alternately using 25% acetonitrile (ACN) in 25 mM ABC and 50% ACN in 25 mM ABC. To reduce and alkylate proteins, the samples were treated with 50 mM DTT in 25 mM ABC for 45 min at 37 °C, followed by treatment with 10 mg/mL IAA in 25 mM ABC for 1 h at room temperature in the dark. Subsequently, the bands were washed twice with 50% ACN in 25 mM ABC. Gel pieces were dehydrated in 100% ACN and subjected to overnight trypsin solution digestion at 37 °C. The resulting peptides were extracted from the gel pieces and dried through vacuum centrifugation.

The obtained peptides were resuspended in 2% ACN with 0.05% trifluoroacetic acid. Peptide identification was performed by tandem mass spectrometry using the UltiMate 3000 RSLCnano System (Thermo-Dionex, Sunnyvale, CA, USA) coupled with microTOF-Q II (Bruker Daltonics, Bremen, Germany). MS data were obtained by online analysis of peptides eluted with a 60 min gradient from 2% to 40% ACN in 0.05% formic acid/water at a flow rate of 300 nl/min. MS/MS (tandem mass spectrometry) data were acquired in a data-dependent manner by targeting three precursor ions with 30 s of dynamic exclusion.

Raw data were internally recalibrated and analyzed using Data Analysis 4.1 software (Bruker Daltonics, Bremen, Germany). The peak lists were searched using an in-house MASCOT server v.2.3.0 (Matrix Science, London, UK) via Protein-Scape 3.0 (Bruker Daltonics, Bremen, Germany) against a non-redundant Swissprot protein database with Green Plants taxonomy restriction. The following search parameters were applied: digestion by trypsin with a maximum of one missed cleavage; precursor and product ion mass tolerance: 20 ppm and 0.05 Da, respectively; carbamidomethyl (C) as a fixed modification; oxidation (M), deamidated (NQ). PSMs were filtered to an FDR of 1% based on the target decoy approach.

### 2.5. RNA Isolation and Quantitative Real Time PCR

Etiolated seedlings of *P. coccineus* were harvested after 8–10 days after sowing when the epicotyl reached a length of about 15 cm. Each epicotyl was dissected into four sections: primary leaves, upper, middle and bottom stem parts. Each seedling fragment, weighing 50–100 mg was immediately frozen in liquid nitrogen. Each sample was ground to powder for RNA isolation, which was performed using Spectrum™ Plant Total RNA Kit (Sigma-Aldrich/Merck, Darmstadt, Germany). Initial preheating of samples with lysis buffer was skipped to avoid starch gelatinization and clogging of the silica-based spin column. About 1 μg of the extracted RNA was transcribed to cDNA using RevertAid First Strand cDNA Synthesis Kit and random hexamer primers (ThermoFisher Scientific, Waltham, MA, USA). The PCR reactions were performed using Illumina Eco™ Real-Time PCR System (5200 Illumina Way, San Diego, CA, USA) with Eco Control software (Eco Real-Time PCR System, version 5.0.16.0) using Real-Time 2× RT PCR Mix SYBR^®^ (A&A Biotechnology, Gdansk, Poland). Reactions were initiated at 95 °C for 5 min, followed by 40 cycles of 10 s at 95 °C, 30 s of annealing (56 °C for GNbP, 61 °C for *LOX2*, 62 °C for *LOX1*, or 54 °C for other primers) and 15 s at 72 °C. Primer sequences were as follow: *GUANINE NUCLEOTIDE-BINDING PROTEIN BETA SUBUNIT-LIKE PROTEIN*, *GNbP* (for: 5′-TGGGCAATTGGACGTTATTAG-3′; rev: 5′-GCCACGGTCTTGAACATAAAA-3′) [20]; *ELONGATION FACTOR 1*, *PvEF1* (for: 5′-CGGGTATGCTGGTGACTTTT-3′; rev: 5′-CACGCTTGAGATCCTTGACA-3′) [21]; *LOX1* (for: 5′-TCTATGCTTAAGTGTTGTATGTGGA-3′; rev: 5′-GCTCCACTTGAAAACCCCAGT-3′); *LOX2* (for: 5′-CAGGTGGTCTTTTCGGTGCT-3′; rev: 5′-CCATTTCCAAGCCCATCAGTT-3′); *LOX3* (for: 5′-AGGGTGTCATTGGAACAGGA-3′; rev: 5′-TCCCTTTTCCATGTCCATCAGG-3′). GeNorm v3.4 [22] was used to calculate the normalization factor for *PvEF4* and *GNbP* reference genes. The normalization factor was then used to calculate the expression levels of the *LOX* genes using the ΔΔCt method [23]. All the results were calibrated to the expression levels of the primary leaves of etiolated seedlings. The experiment was conducted in biological tetraplicate and technical triplicate.

### 2.6. Phylogenetic and Sequence Analysis

The protein sequences of lipoxygenases from *Phaseolus vulgaris* were downloaded from Uniprot database (June2023) [24]. To eliminate fragments of sequences, only those longer than 740 amino acids were selected. Additionally, sequences with higher than 98% pairwise protein sequence similarity to other sequences in the dataset were removed. The phylogenetic analysis was performed using Mega11 version 11.0.10 [25]. Sequences were aligned with the Muscle algorithm under default settings (Figure S4) [26]. For the analysis, we chose the LG substitution model with a gamma distribution of the rate variation among sites (shape parameter = 2) [27], as it was the most suitable for our dataset according to the analysis performed with MEGA version 11.0.10. The tree was constructed with the maximum likelihood algorithm with complete deletion of gaps within the alignment since it provided the most probable tree under the assumed statistical model [28]. Moreover, support for the nodes in the tree was assessed by non-parametric bootstrapping with 500 replicates. The differences in the composition bias among sequences were considered in evolutionary comparisons [29]. The analysis involved 27 amino acid sequences. There were 625 positions in the final dataset. All trees presented in this study were visualized using iTOL [30].

The amino acid sequences of LOX1-6 proteins were aligned with the Clustal omega online tool [31].

## 3. Results

### 3.1. Purification and Identification of the Oxidase from Phaseolus coccineus

**Figure 1:**
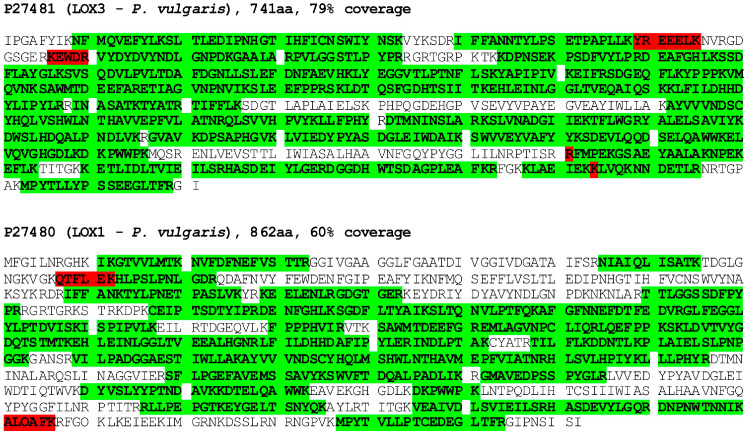
Protein identification by mass spectrometry in bands excised from PAGE gel. Tryptic peptides identified by MS (in green with high and in red with low reliability) are highlighted along the LOX3 (P27481) and LOX1 (P27480) sequences. See Section 2 for details.

As the previously applied approaches were unsuccessful in the purification of the oxidase, currently we tested various chromatographic media and elution conditions to obtain at least partial enrichment of the enzyme in the obtained fractions. The most successful in this respect turned out to be chromatography on DEAE-Sepharose in the citric buffer and the elution of the absorbed proteins with increasing concentrations of arginine. The highest activity of the oxidase was found in fractions eluted with 50 mM arginine when the activity was measured by a standard test in mixed EYL/Toc liposomes. The stabilization of the oxidase by arginine is probably related to its preventing of protein aggregation and increasing their solubility [32,33]. The fractions with the highest oxidase activity were concentrated by ultrafiltration and subject to mass spectrometry (MS) analysis. The obtained results (Figure S1) show that the protein with the best fit was lipoxygenase from Phaseolus vulgaris (accession number P27481). The other lipoxygenases identified were the enzymes corresponding to those from *P. vulgaris* (P27480) and from Glycine max. Within the long list of proteins, there were no other oxidases or any other oxidoreductases which could oxidize tocopherols. Next, the fractions analyzed above were subject to SDS-PAGE and the bands, corresponding with the molecular mass to lipoxygenases (80–100 kDa), were excised; after protein extraction, they were analyzed again by MS. The results show (Figure S2) that both lipoxygenases of *P. vulgaris* were among the proteins with the best fit even with the higher coverage (80 and 60% for P27481 and P27480, respectively) in this case. The coverage of the obtained peptides along the amino acid sequences of both isoforms of lipoxygenases (LOX3 and LOX1, respectively) is shown in Figure 1. These data indicate that either one or both of the identified isoforms of lipoxygenases may be responsible for the tocopherol oxidase activity.

### 3.2. Analysis of Activity and Expression Profiles of Lipoxygenases in Etiolated Seedlings

**Figure 2:**
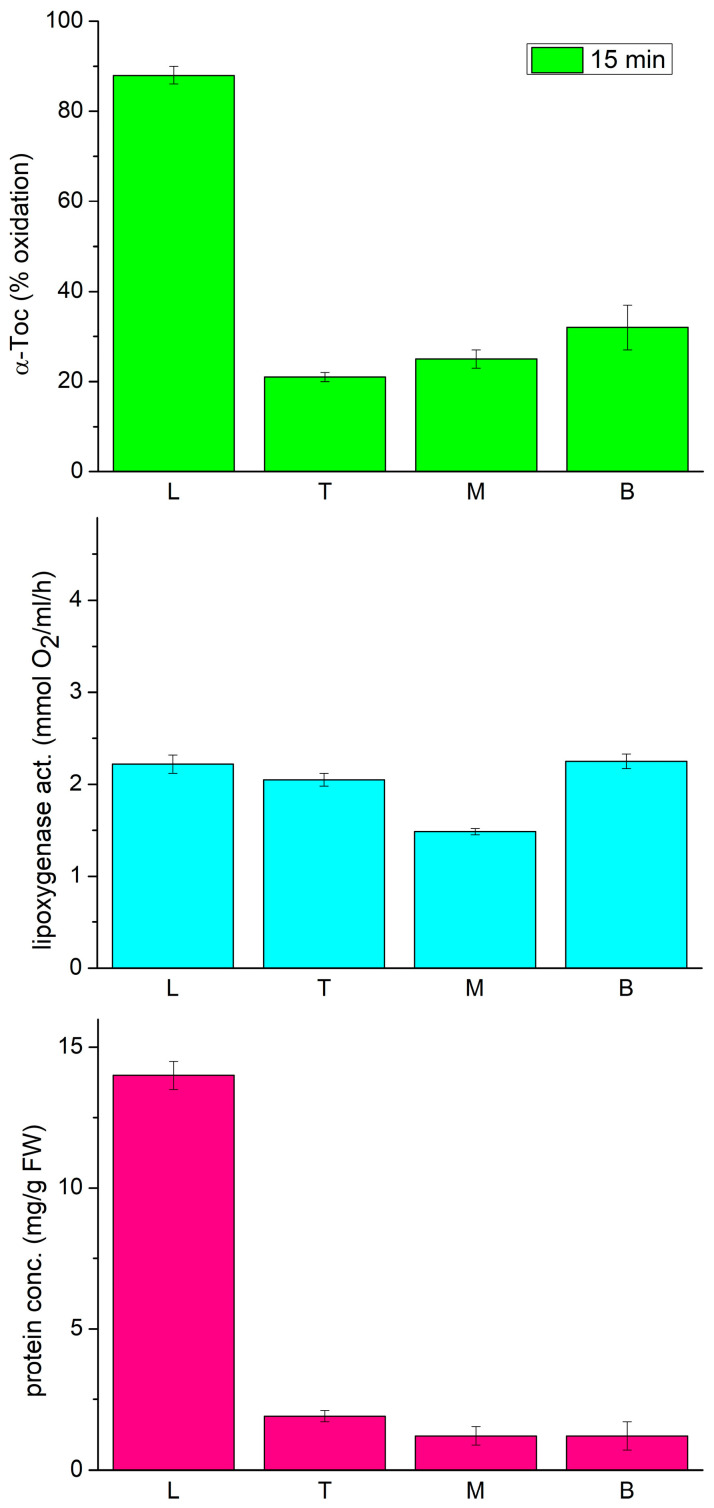
Tocopherol oxidase activity, lipoxygenase activity and protein concentration in extracts from different parts of 12-day-old etiolated seedlings of *P. coccineus*. L—leaves, T—top, M—middle, B—bottom part of the epicotyl.

In the following experiments, we decided to analyze the lipoxygenase and tocopherol oxidase activity, along with the expression profile of three LOX isoforms, LOX1 (Gene Bank accession X63525), LOX2 (U76687), and LOX3 (X63521), in different parts of the etiolated seedlings of P. coccineus. These isoforms were previously reported to be expressed in young seedlings of *P. vulgaris* [34,35,36]. As tocopherol oxidase activity was previously found to depend on the region of the seedlings [18], therefore we used it as a guidance to find which of the LOX isoforms is responsible for the tocopherol oxidase activity. As can be seen in Figure 2, the oxidase activity was considerably higher in the primary leaves than in the epicotyl. Moreover, the Toc oxidase activity correlated well with the total protein content in the investigated parts of the seedlings. On the other hand, the lipoxygenase activity, measured by oxygen consumption with the Clark electrode, was similar in all the parts of the seedlings.

**Figure 3:**
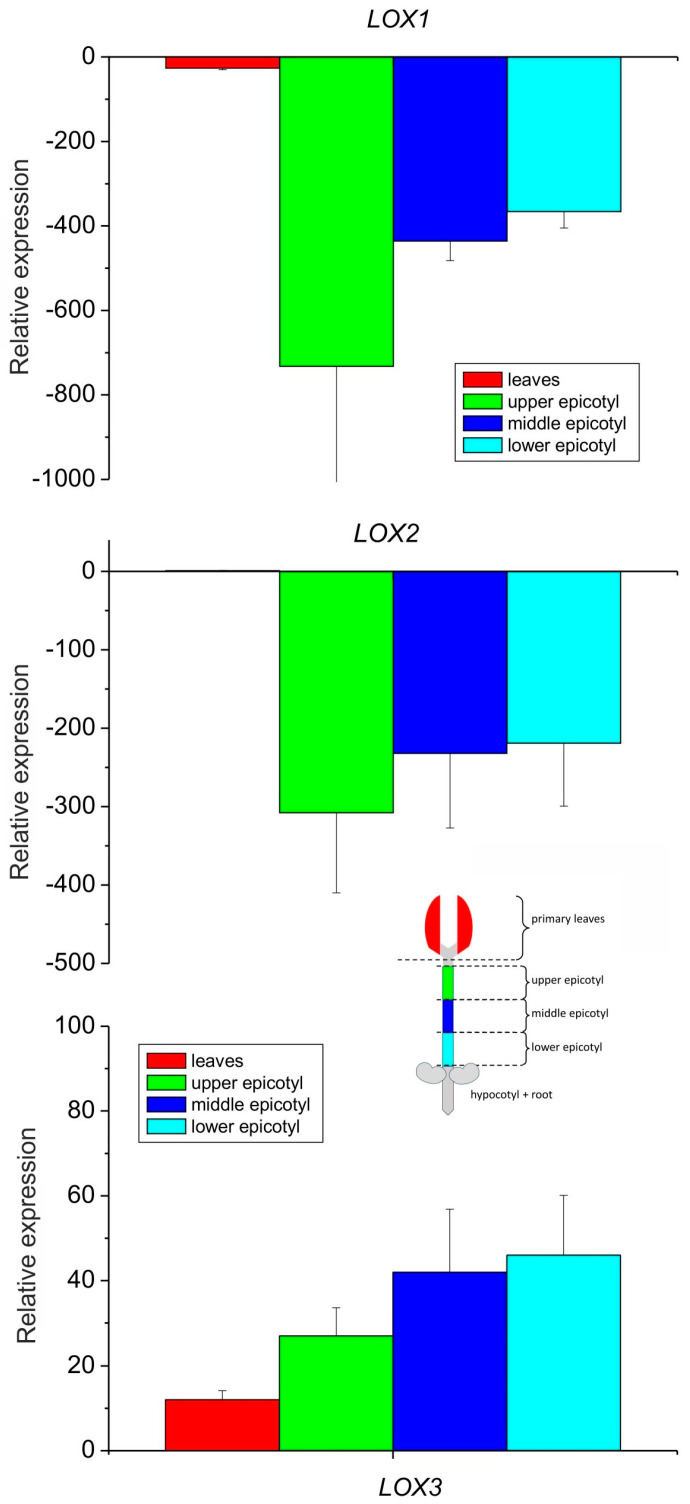
Relative mRNA expression of LOX1, LOX2 and LOX3 genes in different parts of 8–10-day-old etiolated seedlings of *P. coccineus*. See Section 2 for details.

The expression analysis of the three lipoxygenase isoforms showed that the LOX3 expression profile increased gradually from leaves towards the bottom of the epicotyl, while the expression of LOX2 and LOX1 was down-regulated drastically in the epicotyl as compared to the leaves (Figure 3), which is in line with previous observations of generally higher accumulation of LOX2 transcripts in developing organs and a decrease in their content in mature seedling fragments [35]. On the other hand, LOX3 expression was shown to be modulated by various biotic and abiotic factors [37].

**Figure 4:**
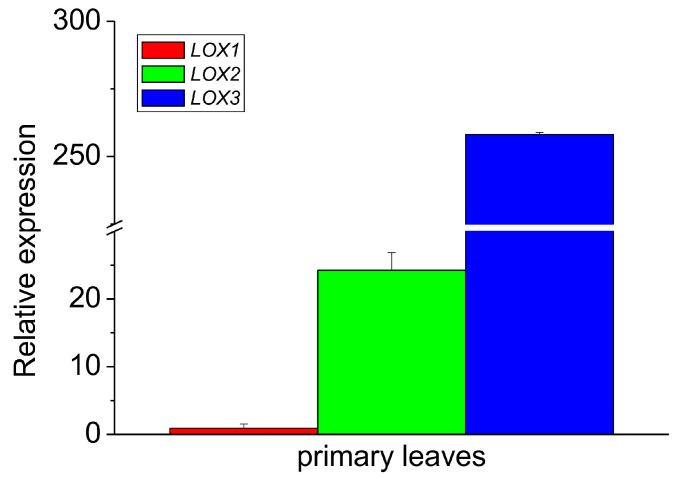
Relative mRNA expression of LOX1, LOX2 and LOX3 genes in the primary leaves of 8–10-day-old etiolated seedlings of *P. coccineus*. See Section 2 for details.

When the relative expression of all the isoforms was compared in the leaves only, the expression of LOX3 was about ten times higher than that of LOX2, while the expression of LOX2 was over 25 times higher than that of LOX1 (Figure 4). These data indicate that among the analyzed isoforms, the expression of LOX1 and LOX2 was negligible in the epicotyl, while in the leaves, the expression of LOX3 dominated with the small contribution of other LOX isoforms. The expression profile of LOX3 in different parts of the epicotyl showed some correlation with the activities of lipoxygenase and tocopherol oxidase, while in the leaves the oxidase activity was the highest, in contrast to the expression of LOX3. This might indicate that in the leaves the oxidase activity of the other LOX isoforms contributed and/or the high concentration of other proteins in the leaves may have somehow stimulated the oxidase activity.

Within the seedlings, the content of Tocs was the highest in the primary leaves, while in different parts of the epicotyl it was considerably lower (Table 1). This suggests that the high oxidase activity in the primary leaves did not result in Tocs degradation in these organs.

### 3.3. Biochemical and Physiological Characterization of the Oxidase/Lipoxygenase

**Figure 5:**
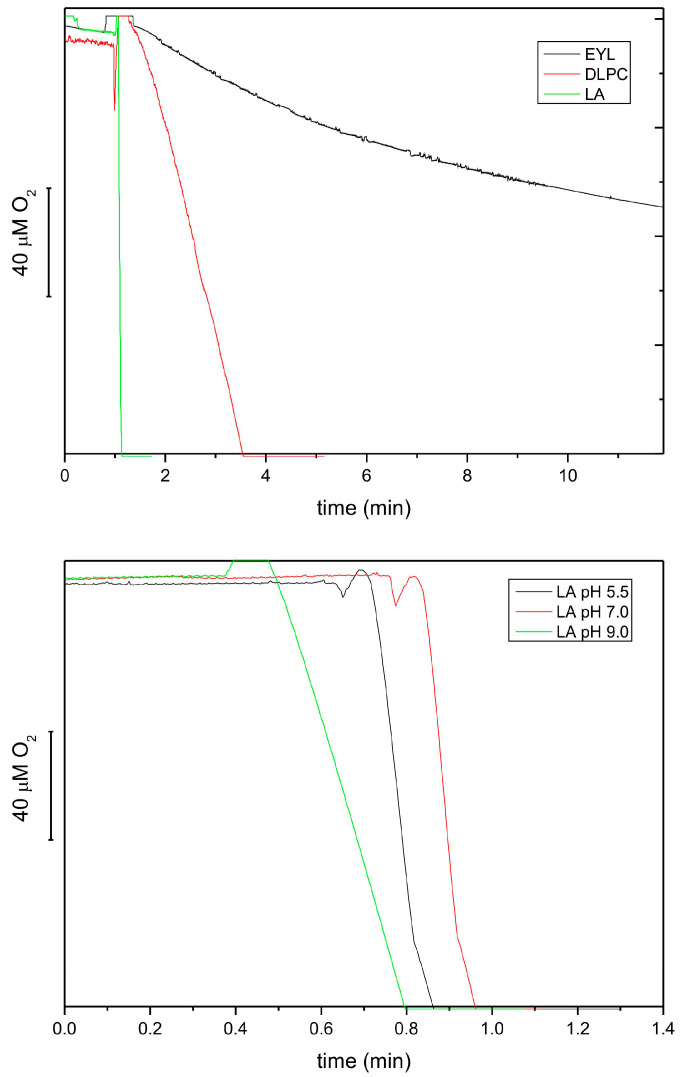
Lipoxygenase activity of extracts from 8-day-old etiolated seedlings of *P. coccineus* using different lipid substrates, followed by oxygen consumption at pH 5.5 (**top**). The activities were 5.67, 0.29 and 0.04 mmol O_2_/mL/h for LA, DLPC and EYL, respectively. The effect of pH on the lipoxygenase activity using LA as a substrate (**bottom**). The following buffers were used: 50 mM citric buffer, 50 mM phosphate buffer and 50 mM borate buffer.

As it was reported in the literature [38,39] that various LOX isoforms show different substrate specificity and pH optimum, we characterized the extract from etiolated seedlings of P. coccineus under this respect. Figure 5 shows that the reaction rate for linoleic acid (LA) was 20 times higher than that for DLPC and ca. 80 times higher than for EYL. These data is in line with previous reports showing that free linoleic acid is a much better substrate than membrane lipids. The relative activity for DLPC and EYL correlated approximately with the LA content in these lipids (100 vs. 15%) (https://www.sigmaaldrich.com, accessed on 6 February 2024). The pH dependence of the reaction showed that the optimum of the reaction was the neutral–acidic range; therefore, the lipoxygenase(s) in the extract could be classified as type 2 [34]. The pH optimum range observed here for the lipoxygenase reaction was similar to that reported for tocopherol oxidase (pH 5.5) [16].

**Figure 6:**
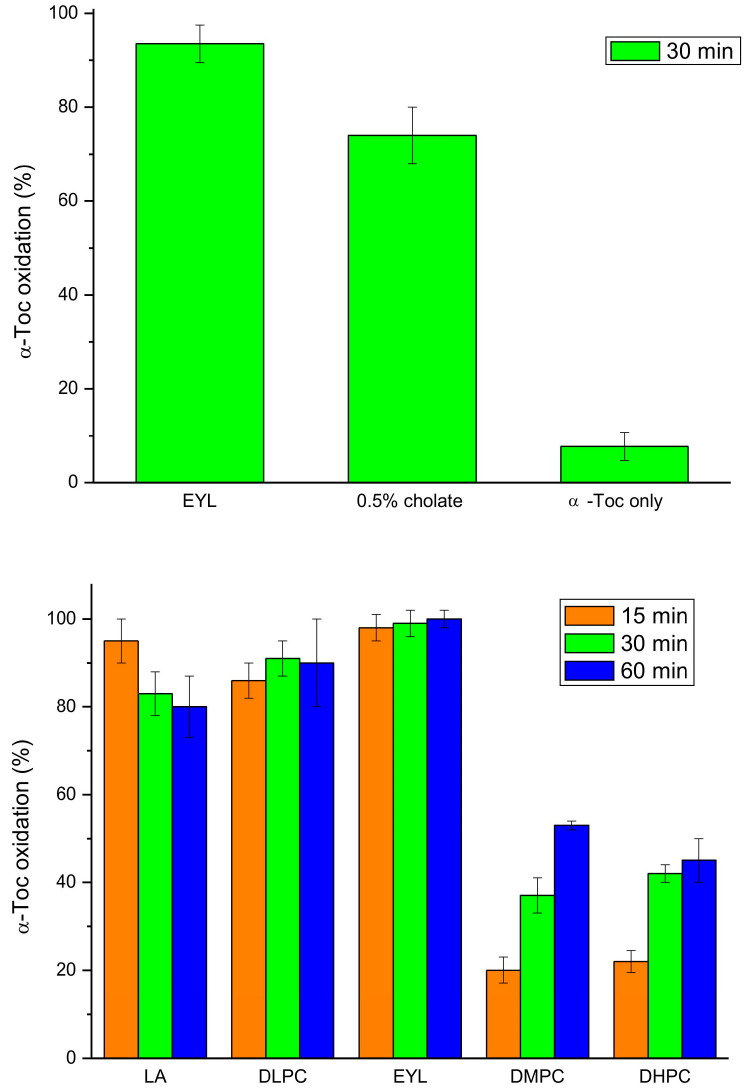
Tocopherol oxidase activity of extract from 9-day-old etiolated seedlings of *P. coccineus* using different lipid systems after the indicated time of reaction. LA—linoleic acid, EYL—egg yolk lecithin, DLPC—dilinoleoyl-phosphatidylcholine, DMPC—dimyristoyl-phosphatidylcholine, DHPC—diheptanoyl-phosphatidylcholine.

Next, we investigated the effect of unsaturated lipids on tocopherol oxidase activity, as it was reported that natural lipids are required for the reaction [16]. We found that incorporation of α-Toc into the EYL liposomes or cholate micelles greatly increased the oxidation activity, as compared to the reaction with α-Toc only, in accordance with previous studies [18] (Figure 6). The reaction in the LA micelles and DLPC liposomes was nearly as fast as in the EYL liposomes, while in those prepared from lipids containing saturated fatty acids (DMPC and DHPC), it was considerably slower but more pronounced that that with α-Toc only (Figure 6). These data indicate that unsaturated lipids are not required for the oxidase reaction but a hydrophobic lipid environment facilitates substrate availability for the enzyme in this reaction.

**Figure 7:**
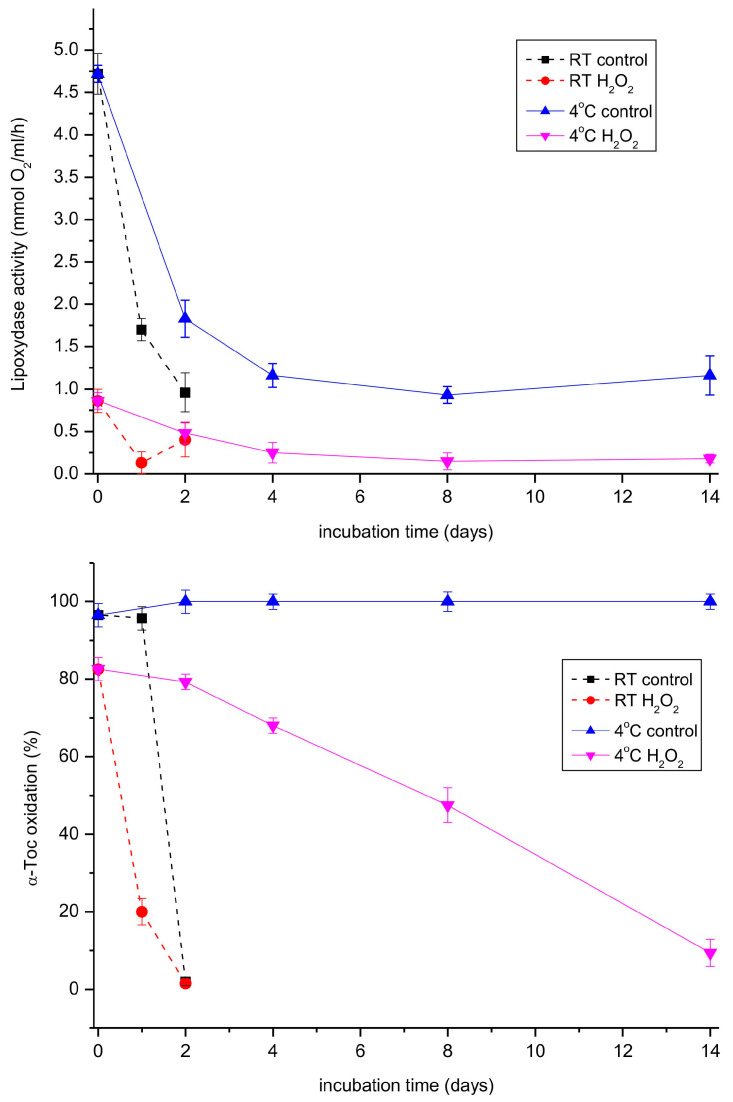
Lipoxygenase and tocopherol oxidase activity in the extract prepared from 11-day-old etiolated seedlings of *P. coccineus* in the course of storage. The extract was stored at room temperature (RT) or 4 °C. Where indicated, hydrogen peroxide was added to 0.006% and the mixture was incubated for 5 min before the measurement.

When both the lipoxygenase and tocopherol oxidase reactions were followed during storage of the extract at room temperature and 4 °C, the activity of the lipoxygenase reaction decreased considerably faster than the oxidase reaction at both temperatures (Figure 7). Interestingly, hydrogen peroxide, a known inhibitor of lipoxygenases [40], inhibited both the investigated reactions, indicating that the same enzyme was most probably responsible for both reactions. We also tested a number of other potential inhibitors of lipoxygenases (quercetin, SHAM, various Fe-chelators) on both reactions, but none of them turned out to be active.

**Figure 8:**
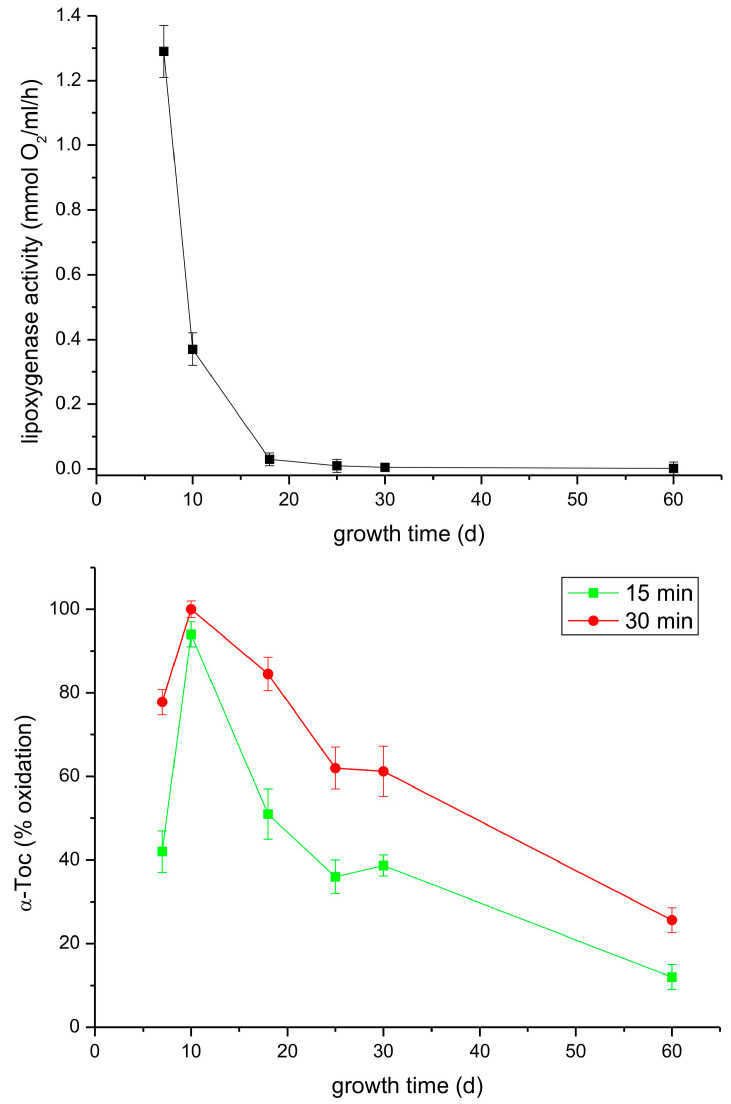
Lipoxygenase and tocopherol oxidase activity of extracts from leaves of light-grown *P. coccineus* of different age. See Section 2 for details.

In the following experiment, we analyzed both the lipoxygenase and tocopherol oxidase reactions in extracts from leaves of light-grown P. coccineus plants during growth for two months. As can be seen in Figure 8, the lipoxygenase activity decreased considerably faster than that of tocopherol oxidase. The reason for the observed differences could be that in the analyzed extract there was a mixture of lipoxygenases whose lipoxygenase activity declines at similar rates but some of the isoforms are not active in Toc oxidation.

### 3.4. Phylogenetic and Sequence Analysis of Lipoxygenases

**Figure 9:**
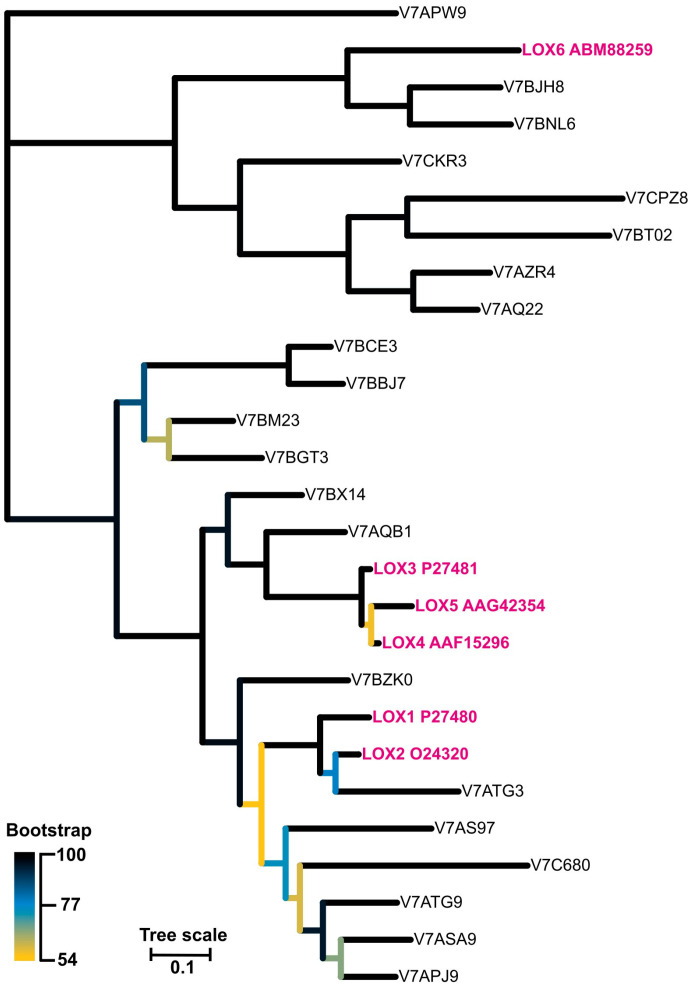
The unrooted phylogenetic tree of lipoxygenase from *Phaseolus vulgaris*. The branches are colour-coded according to the bootstrap value of the nodes. The ‘leaves’ representing isoforms LOX1-6 are marked in pink. The tree with the highest log likelihood (−17,731.26) is shown.

As there are known at least six lipoxygenases (LOX1-6) in *P. vulgaris* characterized biochemically and physiologically [34,35,36,41,42,43], we performed sequence alignment of these enzymes to highlight the differences and similarities within the sequences (Figure S3). LOX3, which is supposed to be the main tocopherol oxidase, differed from the other isoforms by the shortest amino acid polypeptide chain by at least 100 amino acids which was truncated mainly from the N-terminal end of the protein. Currently, it is not possible to decide if this is the reason for the tocopherol oxidase activity of this isoform. Besides the LOX1-6 isoforms, there were 21 other LOX-es found in the *P. vulgaris* genome, described as ‘predicted proteins’. The phylogenetic relations of all these lipoxygenases is shown in Figure 9 and the corresponding sequences in Figure S4. Further studies are required to determine if these genes code different, functional proteins.

## 4. Discussion

Lipoxygenases represent a large group of plant enzymes with especially numerous isoforms found in the Fabaceae family (*Glycine max*, *Pisum sativum*, *Phaseolus vulgaris*) [36,38]. Many of the enzymes have been characterized, particularly in soybean [44,45,46] because of practical aspects as they occur in consumed seeds. Lipoxygenases are found in various plant organs and cellular compartments [39], and they play a variety of physiological functions, e.g., regulate vegetative growth, serve as storage proteins, are engaged in lipid mobilization during germination, are signaling molecules during wounding, herbivore or pathogen attack [47]. Although lipoxygenases are supposed to catalyze only the reaction of lipid peroxidation, a variety of compounds with physiological activity could be formed from lipid hydroperoxides [38,47].

Mass spectrometry analysis revealed that no other redox-active enzymes that could be responsible for the observed tocopherol oxidase activity (peroxidases, polyphenol oxidases, laccases, ascorbate oxidase, etc.) were found in the purified fractions from *P. coccineus*. It should be mentioned here that the extract from *P. coccineus* did not oxidize catechol either at pH 5.5 or 7.5, in the presence or absence of EYL, indicating that tocopherol oxidase does not show polyphenol oxidase (PPO) activity. On the other hand, laccase from *Trametes versicolor* (Sigma-Aldrich), belonging to multicopper oxidases (MCO) [48], oxidized α-Toc in the EYL liposomes efficiently. However, the extract from *P. coccineus* did not oxidize syringaldazine in contrast to the laccase. The latter reaction is the indicator of laccase and peroxidase (in the presence of H_2_O_2_) activity [49]. Moreover, ascorbate oxidase from *Cucurbita* sp. (Sigma-Aldrich), another MCO, did not show any tocopherol oxidase activity either. It was demonstrated that ascorbate oxidase, besides ascorbate, could also accept catechols and polyphenols as substrates, at least in vitro [50].

The presented data indicate that the tocopherol oxidase reaction is slower than that of the lipoxygenase reaction and both reactions seems to be independent, as the latter proceeds in liposomes and micelles in the absence of unsaturated lipids. It might be also an enzymatic side-reaction of lipoxygenase which does not require typical lipoxygenase activity, meaning that the enzyme could be inactivated in the lipoxygenase activity but is still active as tocopherol oxidase. This can be concluded from the faster decline of lipoxygenase activity than that of tocopherol oxidase activity, which can be observed in Figure 7 and Figure 8.

Our data indicate that LOX3 was responsible for at least the majority of the enigmatic tocopherol oxidase activity in etiolated seedlings of *P. coccineus*. LOX3 was the only active isoform in the epicotyls and the dominating one in the primary leaves, where LOX1 and LOX2 could also have contributed. Although *LOX2* expression was higher than that of *LOX1* in the leaves of etiolated seedlings, LOX2 was not detected in MS analysis of the extract. The sequences of LOX1 and LOX2 were evidently different in the regions determined by MS (Figure S3); thus, misidentification can be excluded. Another indication against the contribution of LOX2 to the tocopherol oxidase activity was the inactivity of the seed extract from *P. coccineus* in Toc oxidation, where the lipoxygenase activity was high. LOX2 is the only isoform known to occur in seeds of the closely related *P. vulgaris* [47]. LOX3 was isolated from the extracellular space of hypocotyls of etiolated *P. vulgaris* seedlings and is supposed to be an apoplastic, plasma membrane-bound protein [36]. The protein showed a pH optimum at pH 6.0; thus, in a similar range as the lipoxygenase(s) investigated in the current research. The up-regulation of pLOX3/P87-LOX transcripts in the leaves infected with *Pseudomonas syringa* pv. *phaseolicola* or *P. fluorescens* suggests an involvement of LOX3 in defence mechanisms [51]. If LOX3 in *P. coccineus* is present in the apoplast as well, the role of its tocopherol oxidase activity remains elusive, as Tocs are found within the cell mainly, if not exclusively, in chloroplasts [52,53]; thus, in a different compartment. Nevertheless, taking into account that there were some reports on Tocs detection in extraplastidic membranes [54], it cannot be excluded that Tocs also occur in the plasma membrane. On the other hand, localisation of LOX3 within the cell is also possible. Interestingly, α-tocopherolquinone, the product of α-Toc oxidation by the oxidase, was found to be an insect feeding stimulant on the leaf surface of *Populus* [55], therefore it must be an extracellular metabolite. It is also probable that α-tocopherolquinone plays a signaling role in plant metabolism. The tocopherol oxidase activity could be revealed during accidental tissue damage or food processing (seed grinding), when mixing of cellular compartments takes place, leading to the Toc degradation. Moreover, tocopherol oxidase activity could also be responsible for γ-tocopherol degradation observed in young runner bean leaves [19].

## 5. Conclusions

Purification of extracts from runner bean (*Phaseolus coccineus*) etiolated seedlings, followed by mass spectrometry analysis of proteins showed that the enzyme responsible for tocopherol oxidation activity is lipoxygenase. Biochemical analysis of the enzyme activity along with the expression profile of three *LOX* isoforms (*LOX1*, *LOX2*, *LOX3*) in various parts of etiolated seedlings revealed that *LOX3* was the major isoform expressed in epicotyls, indicating that this isoforms is responsible for the tocopherol oxidation activity. The experiments performed in the model systems showed that unsaturated lipids were not required for the tocopherol oxidase activity but that lipids were necessary to provide the optimal, hydrophobic environment of the substrate for the reaction. As LOX3 in *P. vulgaris* was shown to occur in the apoplast, where Tocs were not reported to occur, the question as to the physiological function of LOX3 in tocopherol oxidation activity in *P. coccineus* remains currently unknown.

## Figures and Tables

**Table 1:** The content of tocopherols in various parts of 8–10-day-old etiolated seedlings of *P. coccineus*. The data are means ± SE, *n* = 4.

Organ	α-Toc (µg/g FW)	γ-Toc (µg/g FW)	α-Toc/γ-Toc
Primary leaves	4.7 ± 0.9	14.5 ± 6.5	0.32
Epicotyl (top)	1.8 ± 0.1	1.5 ± 0.8	1.20
Epicotyl (middle)	1.3 ± 0.1	0.8 ± 0.2	1.62
Epicotyl (bottom)	1.2 ± 0.4	3.4 ± 2.0	0.35

## Data Availability

The original contributions presented in the study are included in the article and Supplementary Material; further inquiries can be directed to the corresponding authors.

## Supplementary Materials

The following supporting information can be downloaded at: https://www.mdpi.com/article/10.3390/antiox13030301/s1, Figure S1: MS analysis of proteins in the tocopherol oxidase fraction after DEAE-Sepharose chromatography. See Section 2 for details; Figure S2: MS analysis of proteins extracted from bands corresponding to lipoxygenase in the PAGE gel. The sample subject for electrophoresis was the fraction analysed in Figure S1; Figure S3: The alignment of amino acid sequences of LOX1-6 proteins from *Phaseolus vulgaris*. Residues are colour-coded according to the chemical properties of the residues. The differences in the peptides found in MS analysis between LOX1 and LOX2 are highlighted in yellow; Figure S4: The aligned sequences of lipoxygenase from *Phaseolus vulgaris* used for a phylogenetic analysis.
