# Supplementary material for: Lipoxygenase LOX3 Is the Enigmatic Tocopherol Oxidase in Runner Bean (Phaseolus coccineus)
Source: Antioxidants (Basel). 2024 Feb 29;13(3):301. doi: 10.3390/antiox13030301 (PMC10967512; doi:10.3390/antiox13030301)
Supplement: Supplementary file 1 [file antioxidants-13-00301-s001.zip › Fig. S1 solution.pdf]

SwissProt Green Plant

| Accession | Description | Score | Coverage | #Peptides | MW[kDa] | calc. pI |
|---|---|---|---|---|---|---|
| **P27481** | **Linoleate 9S-lipoxygenase (Fragment) OS=Phaseolus vulgaris** | **1314.45** | **44.40 %** | **26** | **84.1** | **6.58** |
| P27322 | Heat shock cognate 70 kDa protein 2 OS=Solanum lycopersicum | 1696.19 | 47.98 % | 24 | 70.7 | 5.19 |
| P22953 | Probable mediator of RNA polymerase II transcription subunit 37e OS=Arabidopsis thaliana | 1613.36 | 34.41 % | 20 | 71.3 | 5.12 |
| P29357 | Chloroplast envelope membrane 70 kDa heat shock-related protein OS=Spinacia oleracea | 1363.43 | 37.83 % | 17 | 71.7 | 5.48 |
| P24629 | Heat shock cognate 70 kDa protein 1 OS=Solanum lycopersicum | 1081.55 | 35.69 % | 16 | 71.2 | 5.26 |
| Q01390 | Sucrose synthase OS=Vigna radiata var. radiata | 499.37 | 20.37 % | 16 | 92.0 | 6.51 |
| P13708 | Sucrose synthase OS=Glycine max | 423.90 | 19.38 % | 15 | 92.2 | 6.48 |
| O23755 | Elongation factor 2 OS=Beta vulgaris | 670.75 | 19.81 % | 14 | 93.7 | 6.30 |
| Q02028 | Stromal 70 kDa heat shock-related protein, chloroplastic OS=Pisum sativum | 655.61 | 21.95 % | 14 | 75.5 | 5.35 |
| Q9FGX1 | ATP-citrate synthase beta chain protein 2 OS=Arabidopsis thaliana | 776.97 | 25.99 % | 13 | 65.8 | 7.64 |
| **P38417** | **Linoleate 9S-lipoxygenase-4 OS=Glycine max** | **551.09** | **20.16 %** | **13** | **96.5** | **6.04** |
| A4GG89 | Ribulose bisphosphate carboxylase large chain OS=Phaseolus vulgaris | 518.25 | 26.68 % | 12 | 52.8 | 6.39 |
| Q9LTX9 | Heat shock 70 kDa protein 7, chloroplastic OS=Arabidopsis thaliana | 530.00 | 17.69 % | 11 | 76.9 | 5.30 |
| Q93VT8 | ATP-citrate synthase beta chain protein 1 OS=Oryza sativa subsp. japonica | 617.85 | 20.89 % | 11 | 66.0 | 7.68 |
| **P27480** | **Linoleate 9S-lipoxygenase 1 OS=Phaseolus vulgaris** | **225.20** | **10.67 %** | **9** | **97.1** | **6.96** |
| Q43155 | Ferredoxin-dependent glutamate synthase, chloroplastic OS=Spinacia oleracea | 267.04 | 6.99 % | 9 | 165.3 | 6.19 |
| Q01899 | Heat shock 70 kDa protein, mitochondrial OS=Phaseolus vulgaris | 218.68 | 13.93 % | 8 | 72.5 | 6.20 |
| Q9AWB2 | Sulfite reductase [ferredoxin], chloroplastic (Fragment) OS=Glycine max | 213.93 | 15.53 % | 8 | 63.8 | 9.06 |
| Q05045 | Chaperonin CPN60-1, mitochondrial OS=Cucurbita maxima | 270.49 | 14.26 % | 8 | 61.0 | 5.77 |
| O49169 | Elongation factor 1-alpha OS=Manihot esculenta | 290.87 | 16.93 % | 7 | 49.3 | 9.13 |
| P07219 | Phaseolin, alpha-type OS=Phaseolus vulgaris | 200.89 | 19.27 % | 7 | 49.2 | 5.38 |
| Q69RJ0 | Ferredoxin-dependent glutamate synthase, chloroplastic OS=Oryza sativa subsp. japonica | 201.98 | 4.77 % | 7 | 174.9 | 6.84 |
| P35007 | Adenosylhomocysteinase OS=Catharanthus roseus | 208.35 | 15.26 % | 7 | 53.2 | 5.88 |
| P50249 | Adenosylhomocysteinase OS=Phalaenopsis sp. | 191.61 | 14.64 % | 7 | 53.1 | 6.14 |
| P32112 | Adenosylhomocysteinase OS=Triticum aestivum | 216.26 | 14.23 % | 7 | 53.4 | 5.92 |
| P29185 | Chaperonin CPN60-1, mitochondrial OS=Zea mays | 228.85 | 14.04 % | 7 | 61.2 | 5.85 |
| Q8L7B5 | Chaperonin CPN60-like 1, mitochondrial OS=Arabidopsis thaliana | 222.55 | 12.48 % | 7 | 61.9 | 6.68 |
| P25698 | Elongation factor 1-alpha OS=Glycine max | 293.54 | 13.65 % | 6 | 49.3 | 9.06 |
| Q9S9N1 | Heat shock 70 kDa protein 5 OS=Arabidopsis thaliana | 301.75 | 15.63 % | 6 | 70.9 | 5.41 |
| Q40079 | V-type proton ATPase subunit B 2 OS=Hordeum vulgare | 158.21 | 12.84 % | 6 | 53.7 | 5.24 |
| P24095 | Seed linoleate 9S-lipoxygenase OS=Glycine max | 249.59 | 6.25 % | 6 | 96.8 | 6.15 |
| P02853 | Phaseolin, beta-type OS=Phaseolus vulgaris | 219.46 | 17.10 % | 6 | 47.5 | 5.44 |
| P80607 | Alpha-1,4-glucan-protein synthase [UDP-forming] OS=Zea mays | 136.41 | 17.03 % | 6 | 41.2 | 6.13 |
| O49485 | D-3-phosphoglycerate dehydrogenase 1, chloroplastic OS=Arabidopsis thaliana | 215.56 | 10.61 % | 6 | 63.3 | 6.58 |
| Q39857 | Xyloglucan endotransglucosylase/hydrolase 1 OS=Glycine max | 223.60 | 21.96 % | 6 | 34.4 | 8.19 |
| Q42962 | Phosphoglycerate kinase, cytosolic OS=Nicotiana tabacum | 166.53 | 16.96 % | 5 | 42.3 | 5.97 |
| P29409 | Phosphoglycerate kinase, chloroplastic (Fragment) OS=Spinacia oleracea | 238.68 | 14.32 % | 5 | 45.5 | 6.16 |
| Q03684 | Luminal-binding protein 4 OS=Nicotiana tabacum | 191.28 | 7.20 % | 5 | 73.5 | 5.19 |
| P46257 | Fructose-bisphosphate aldolase, cytoplasmic isozyme 2 OS=Pisum sativum | 381.72 | 17.55 % | 5 | 38.5 | 7.25 |
| P0CG84 | Polyubiquitin (Fragment) OS=Nicotiana sylvestris | 198.63 | 62.33 % | 5 | 42.2 | 7.58 |
| Q9FLW9 | Plastidial pyruvate kinase 2 OS=Arabidopsis thaliana | 174.31 | 8.64 % | 5 | 63.5 | 7.03 |
| P09439 | Seed linoleate 9S-lipoxygenase-2 OS=Glycine max | 272.50 | 5.90 % | 5 | 97.1 | 6.74 |
| P43210 | Delta-aminolevulinic acid dehydratase, chloroplastic OS=Glycine max | 122.47 | 14.81 % | 5 | 45.0 | 7.09 |
| Q9SF16 | T-complex protein 1 subunit eta OS=Arabidopsis thaliana | 145.80 | 10.59 % | 5 | 59.7 | 6.39 |
| Q940P8 | T-complex protein 1 subunit beta OS=Arabidopsis thaliana | 133.87 | 11.95 % | 5 | 57.2 | 5.87 |
| Q8LNZ5 | Probable xyloglucan endotransglucosylase/hydrolase protein B OS=Phaseolus angularis | 134.01 | 14.68 % | 5 | 34.1 | 8.76 |
| P49034 | Sucrose synthase OS=Alnus glutinosa | 172.19 | 5.73 % | 5 | 91.6 | 6.81 |
| Q5QMN3 | DEAD-box ATP-dependent RNA helicase 20 OS=Oryza sativa subsp. japonica | 151.31 | 11.13 % | 5 | 54.6 | 8.27 |
| P05088 | Erythroagglutinating phytohemagglutinin OS=Phaseolus vulgaris | 159.17 | 25.45 % | 5 | 29.7 | 5.38 |
| O80377 | 40S ribosomal protein SA OS=Daucus carota | 190.84 | 10.44 % | 4 | 32.4 | 5.02 |
| P54765 | GTP-binding nuclear protein Ran1A (Fragment) OS=Lotus japonicus | 115.66 | 17.70 % | 4 | 23.8 | 7.12 |
| P46523 | ATP-dependent Clp protease ATP-binding subunit clpA homolog, chloroplastic (Fragment) OS | 124.69 | 5.95 % | 4 | 97.3 | 6.18 |
| Q9SMQ6 | Ras-related protein RABA4b OS=Arabidopsis thaliana | 126.96 | 17.86 % | 4 | 24.4 | 6.09 |
| Q42560 | Aconitate hydratase 1 OS=Arabidopsis thaliana | 67.14 | 4.45 % | 4 | 98.1 | 6.40 |
| Q6YZX6 | Putative aconitate hydratase, cytoplasmic OS=Oryza sativa subsp. japonica | 86.75 | 4.34 % | 4 | 98.0 | 6.01 |
| O04865 | Phospholipase D alpha 1 OS=Vigna unguiculata | 116.59 | 4.94 % | 4 | 91.5 | 5.85 |
| Q96558 | UDP-glucose 6-dehydrogenase 1 OS=Glycine max | 120.78 | 10.00 % | 4 | 52.9 | 6.04 |
| Q9LF33 | UDP-glucose 6-dehydrogenase 3 OS=Arabidopsis thaliana | 130.05 | 10.63 % | 4 | 53.1 | 6.04 |
| Q43468 | Hsp70-Hsp90 organizing protein 1 OS=Glycine max | 196.28 | 7.87 % | 4 | 64.1 | 5.69 |
| P93254 | S-adenosylmethionine synthase OS=Mesembryanthemum crystallinum | 98.36 | 15.82 % | 4 | 42.9 | 5.71 |
| Q9SRT9 | UDP-arabinopyranose mutase 1 OS=Arabidopsis thaliana | 95.78 | 11.48 % | 4 | 40.6 | 5.92 |
| P85413 | Alpha-1,4-glucan-protein synthase [UDP-forming] (Fragments) OS=Phoenix dactylifera | 102.83 | 66.67 % | 4 | 6.8 | 8.16 |
| Q9SN95 | UDP-glucuronic acid decarboxylase 5 OS=Arabidopsis thaliana | 114.31 | 18.48 % | 4 | 38.4 | 7.52 |
| Q84WU2 | Ubiquitin carboxyl-terminal hydrolase 13 OS=Arabidopsis thaliana | 81.75 | 4.57 % | 4 | 130.6 | 5.58 |
| P52780 | Glutamine--tRNA ligase OS=Lupinus luteus | 146.10 | 4.66 % | 4 | 90.4 | 6.76 |
| Q41141 | Pyrophosphate--fructose 6-phosphate 1-phosphotransferase subunit beta OS=Ricinus comm | 140.46 | 8.33 % | 4 | 60.1 | 6.64 |
| P49039 | Sucrose synthase OS=Solanum tuberosum | 142.34 | 5.09 % | 4 | 92.5 | 6.40 |
| P24007 | Ribulose bisphosphate carboxylase small chain, chloroplastic OS=Pyrus pyrifolia | 122.73 | 15.85 % | 3 | 20.5 | 8.87 |
| O81221 | Actin OS=Gossypium hirsutum | 76.47 | 8.22 % | 3 | 41.6 | 5.49 |
| Q9SGE0 | UDP-D-apiose/UDP-D-xylose synthase 2 OS=Arabidopsis thaliana | 90.66 | 8.23 % | 3 | 43.8 | 5.87 |
| O82802 | Sulfite reductase 1 [ferredoxin], chloroplastic OS=Nicotiana tabacum | 101.82 | 4.18 % | 3 | 77.9 | 9.04 |
| O80988 | Glycine dehydrogenase (decarboxylating) 2, mitochondrial OS=Arabidopsis thaliana | 116.54 | 3.07 % | 3 | 113.7 | 6.65 |
| O49939 | Peptidyl-prolyl cis-trans isomerase, chloroplastic OS=Spinacia oleracea | 105.99 | 7.35 % | 3 | 49.8 | 5.41 |
| P93400 | Phospholipase D alpha 1 OS=Nicotiana tabacum | 78.15 | 3.34 % | 3 | 91.9 | 5.63 |
| P09559 | Phosphoribulokinase, chloroplastic OS=Spinacia oleracea | 133.47 | 7.46 % | 3 | 45.0 | 6.21 |

| | | | | | | |
|---|---|---|---|---|---|---|
| P42896 | Enolase OS=Ricinus communis | 45.54 | 10.34 % | 3 | 47.9 | 5.78 |
| P31300 | Cysteine synthase, chloroplastic/chromoplastic OS=Capsicum annuum | 93.43 | 12.30 % | 3 | 40.0 | 5.27 |
| P08927 | RuBisCO large subunit-binding protein subunit beta, chloroplastic OS=Pisum sativum | 160.00 | 4.03 % | 3 | 62.9 | 6.04 |
| O82392 | Phosphomethylpyrimidine synthase, chloroplastic OS=Arabidopsis thaliana | 56.46 | 5.75 % | 3 | 71.9 | 6.44 |
| P27774 | Phosphoribulokinase, chloroplastic OS=Mesembryanthemum crystallinum | 71.56 | 7.81 % | 3 | 44.1 | 6.46 |
| Q8W250 | 1-deoxy-D-xylulose 5-phosphate reductoisomerase, chloroplastic OS=Oryza sativa subsp. japo | 120.68 | 8.03 % | 3 | 51.4 | 6.18 |
| Q9XI91 | Eukaryotic translation initiation factor 5A-1 OS=Arabidopsis thaliana | 76.81 | 18.35 % | 3 | 17.3 | 5.76 |
| Q84WV1 | T-complex protein 1 subunit gamma OS=Arabidopsis thaliana | 87.99 | 5.23 % | 3 | 60.3 | 5.77 |
| Q9AXJ4 | Eukaryotic translation initiation factor 5A OS=Manihot esculenta | 74.49 | 18.13 % | 3 | 17.6 | 6.20 |
| P53780 | Cystathionine beta-lyase, chloroplastic OS=Arabidopsis thaliana | 103.47 | 6.03 % | 3 | 50.4 | 7.36 |
| Q9XFS9 | 1-deoxy-D-xylulose 5-phosphate reductoisomerase, chloroplastic OS=Arabidopsis thaliana | 136.59 | 6.29 % | 3 | 51.9 | 7.05 |
| Q9SYM5 | Trifunctional UDP-glucose 4,6-dehydratase/UDP-4-keto-6-deoxy-D-glucose 3,5-epimerase/UD | 86.14 | 5.38 % | 3 | 75.3 | 7.28 |
| Q8W4M5 | Pyrophosphate--fructose 6-phosphate 1-phosphotransferase subunit beta 1 OS=Arabidopsis | 127.23 | 9.19 % | 3 | 61.4 | 6.10 |
| O23735 | Cysteine synthase OS=Brassica juncea | 144.93 | 9.88 % | 3 | 34.1 | 5.59 |
| Q42806 | Pyruvate kinase, cytosolic isozyme OS=Glycine max | 97.48 | 5.68 % | 3 | 55.3 | 7.56 |
| O22718 | ATP-citrate synthase alpha chain protein 2 OS=Arabidopsis thaliana | 136.10 | 6.38 % | 3 | 46.7 | 5.27 |
| P51061 | Phosphoenolpyruvate carboxylase OS=Glycine max | 79.37 | 5.48 % | 3 | 110.7 | 6.47 |
| O49835 | Phenylalanine ammonia-lyase 1 OS=Lithospermum erythrorhizon | 51.43 | 5.35 % | 3 | 77.5 | 6.00 |
| B9DGT7 | Tubulin alpha-2 chain OS=Arabidopsis thaliana | 88.64 | 7.33 % | 3 | 49.5 | 5.06 |
| Q42662 | 5-methyltetrahydropteroyltriglutamate--homocysteine methyltransferase OS=Plectranthus s | 100.35 | 2.49 % | 2 | 84.5 | 6.51 |
| P09044 | Glyceraldehyde-3-phosphate dehydrogenase B, chloroplastic (Fragment) OS=Nicotiana tabac | 56.92 | 4.57 % | 2 | 47.4 | 8.57 |
| Q01525 | 14-3-3-like protein GF14 omega OS=Arabidopsis thaliana | 65.77 | 6.18 % | 2 | 29.1 | 4.79 |
| P46266 | 14-3-3-like protein OS=Pisum sativum | 71.91 | 7.31 % | 2 | 29.3 | 4.79 |
| P00865 | Ribulose bisphosphate carboxylase small chain 1, chloroplastic OS=Glycine max | 87.95 | 14.04 % | 2 | 20.1 | 8.66 |
| A6MML4 | ATP synthase subunit beta, chloroplastic OS=Dioscorea elephantipes | 59.23 | 4.64 % | 2 | 53.4 | 5.39 |
| P36181 | Heat shock cognate protein 80 OS=Solanum lycopersicum | 49.88 | 2.86 % | 2 | 80.1 | 5.03 |
| P37829 | Fructokinase OS=Solanum tuberosum | 60.25 | 7.52 % | 2 | 33.7 | 5.71 |
| Q40466 | Eukaryotic initiation factor 4A-13 (Fragment) OS=Nicotiana tabacum | 55.17 | 5.35 % | 2 | 40.2 | 5.33 |
| P02873 | Alpha-amylase inhibitor 1 OS=Phaseolus vulgaris | 61.92 | 10.98 % | 2 | 27.2 | 5.24 |
| Q6ESI7 | Tripeptidyl-peptidase 2 OS=Oryza sativa subsp. japonica | 31.07 | 1.32 % | 2 | 148.5 | 6.58 |
| P10933 | Ferredoxin--NADP reductase, leaf isozyme, chloroplastic OS=Pisum sativum | 46.50 | 6.39 % | 2 | 40.2 | 8.40 |
| O04977 | Ferredoxin--NADP reductase, leaf-type isozyme, chloroplastic OS=Nicotiana tabacum | 42.57 | 4.70 % | 2 | 40.4 | 8.21 |
| O04130 | D-3-phosphoglycerate dehydrogenase 2, chloroplastic OS=Arabidopsis thaliana | 97.68 | 3.21 % | 2 | 66.4 | 6.13 |
| Q01289 | Protochlorophyllide reductase, chloroplastic OS=Pisum sativum | 96.67 | 7.27 % | 2 | 42.9 | 9.04 |
| Q96372 | Cell division cycle protein 48 homolog OS=Capsicum annuum | 83.31 | 2.24 % | 2 | 89.3 | 5.20 |
| Q94A28 | Aconitate hydratase 3, mitochondrial OS=Arabidopsis thaliana | 48.24 | 2.41 % | 2 | 108.4 | 7.15 |
| Q00834 | Cysteine synthase OS=Spinacia oleracea | 131.74 | 7.69 % | 2 | 34.2 | 5.62 |
| Q01517 | Fructose-bisphosphate aldolase 2, chloroplastic OS=Pisum sativum | 86.96 | 4.87 % | 2 | 37.8 | 5.59 |
| Q40255 | Probable aldehyde dehydrogenase OS=Linum usitatissimum | 51.29 | 3.45 % | 2 | 61.0 | 7.28 |
| O24457 | Pyruvate dehydrogenase E1 component subunit alpha-3, chloroplastic OS=Arabidopsis thalia | 71.92 | 5.37 % | 2 | 47.1 | 7.49 |
| O20252 | Sedoheptulose-1,7-bisphosphatase, chloroplastic OS=Spinacia oleracea | 61.97 | 5.94 % | 2 | 42.1 | 6.20 |
| P29356 | Fructose-bisphosphate aldolase, cytoplasmic isozyme OS=Spinacia oleracea | 75.78 | 4.20 % | 2 | 38.4 | 6.28 |
| P46283 | Sedoheptulose-1,7-bisphosphatase, chloroplastic OS=Arabidopsis thaliana | 53.32 | 7.63 % | 2 | 42.4 | 6.57 |
| Q42669 | Aconitate hydratase (Fragment) OS=Cucumis melo var. conomon | 89.39 | 2.62 % | 2 | 83.2 | 6.80 |
| Q9STH1 | Hsp70-Hsp90 organizing protein 3 OS=Arabidopsis thaliana | 117.02 | 3.23 % | 2 | 63.7 | 6.32 |
| P31239 | 1-aminocyclopropane-1-carboxylate oxidase OS=Pisum sativum | 72.50 | 8.52 % | 2 | 36.0 | 5.19 |
| Q9SIU0 | NAD-dependent malic enzyme 1, mitochondrial OS=Arabidopsis thaliana | 32.02 | 4.65 % | 2 | 69.6 | 5.45 |
| P56821 | Eukaryotic translation initiation factor 3 subunit B OS=Nicotiana tabacum | 64.20 | 2.36 % | 2 | 82.5 | 5.02 |
| Q9SU63 | Aldehyde dehydrogenase family 2 member B4, mitochondrial OS=Arabidopsis thaliana | 115.49 | 3.53 % | 2 | 58.6 | 7.46 |
| O48593 | Asparagine--tRNA ligase, chloroplastic/mitochondrial OS=Arabidopsis thaliana | 79.46 | 2.82 % | 2 | 63.7 | 6.05 |
| P08824 | RuBisCO large subunit-binding protein subunit alpha (Fragment) OS=Ricinus communis | 73.85 | 4.24 % | 2 | 52.3 | 4.87 |
| Q9ZPI1 | Lysine--tRNA ligase OS=Arabidopsis thaliana | 45.57 | 2.40 % | 2 | 70.8 | 6.24 |
| P37221 | NAD-dependent malic enzyme 62 kDa isoform, mitochondrial OS=Solanum tuberosum | 37.53 | 4.31 % | 2 | 69.9 | 5.94 |
| Q5Z5B2 | Protein argonaute 1D OS=Oryza sativa subsp. japonica | 38.85 | 1.73 % | 2 | 115.9 | 9.03 |
| Q9LIR4 | Dihydroxy-acid dehydratase, chloroplastic OS=Arabidopsis thaliana | 71.79 | 2.63 % | 2 | 64.9 | 6.23 |
| F4JVN6 | Tripeptidyl-peptidase 2 OS=Arabidopsis thaliana | 63.70 | 2.03 % | 2 | 152.3 | 6.07 |
| O81154 | Cysteine synthase OS=Solanum tuberosum | 133.99 | 7.38 % | 2 | 34.3 | 6.62 |
| P29038 | L-lactate dehydrogenase OS=Zea mays | 89.02 | 7.63 % | 2 | 38.5 | 8.87 |
| P85984 | Profilin (Fragments) OS=Beta vulgaris | 71.98 | 100.00 % | 2 | 2.5 | 4.23 |
| O23627 | Glycine--tRNA ligase 1, mitochondrial OS=Arabidopsis thaliana | 41.62 | 3.16 % | 2 | 81.9 | 7.01 |
| Q5Z7L0 | Beta-galactosidase 9 OS=Oryza sativa subsp. japonica | 95.24 | 2.66 % | 2 | 78.2 | 7.65 |
| Q9S7C0 | Heat shock 70 kDa protein 14 OS=Arabidopsis thaliana | 44.76 | 3.13 % | 2 | 91.7 | 5.25 |
| P55964 | Pyruvate kinase isozyme G, chloroplastic (Fragment) OS=Ricinus communis | 99.93 | 4.55 % | 2 | 46.0 | 6.58 |
| Q2QKB3 | Splicing factor U2af large subunit A OS=Triticum aestivum | 76.20 | 2.88 % | 2 | 67.9 | 8.29 |
| Q9FG38 | Sorting nexin 1 OS=Arabidopsis thaliana | 117.00 | 4.23 % | 2 | 46.5 | 7.24 |
| Q42800 | 4-hydroxy-tetrahydrodipicolinate synthase, chloroplastic OS=Glycine max | 54.41 | 4.82 % | 2 | 36.4 | 6.32 |
| P51074 | Annexin-like protein RJ4 OS=Fragaria ananassa | 191.70 | 6.37 % | 2 | 35.6 | 7.23 |
| Q9XEA8 | Cysteine synthase OS=Oryza sativa subsp. japonica | 89.72 | 7.08 % | 2 | 34.3 | 5.41 |
| Q93Z53 | Plastidial pyruvate kinase 3, chloroplastic OS=Arabidopsis thaliana | 74.30 | 4.73 % | 2 | 62.6 | 7.81 |
| O82013 | 17.3 kDa class II heat shock protein OS=Solanum peruvianum | 47.57 | 16.77 % | 2 | 17.3 | 6.80 |
| Q07176 | Mitogen-activated protein kinase homolog MMK1 OS=Medicago sativa | 69.08 | 6.46 % | 2 | 44.4 | 5.85 |
| Q43621 | Glutathione reductase, cytosolic OS=Pisum sativum | 50.22 | 5.82 % | 2 | 53.9 | 7.01 |
| P21342 | Pyrophosphate--fructose 6-phosphate 1-phosphotransferase subunit alpha OS=Solanum tube | 32.57 | 5.03 % | 2 | 67.3 | 7.43 |
| Q94K05 | T-complex protein 1 subunit theta OS=Arabidopsis thaliana | 72.54 | 4.74 % | 2 | 58.9 | 5.35 |
| O04905 | UMP-CMP kinase 3 OS=Arabidopsis thaliana | 92.95 | 8.42 % | 2 | 22.5 | 6.01 |
| P93394 | Uracil phosphoribosyltransferase OS=Nicotiana tabacum | 55.22 | 11.16 % | 2 | 24.2 | 7.03 |
| O48659 | Spermidine synthase 2 OS=Hyoscyamus niger | 52.57 | 8.44 % | 2 | 33.9 | 5.11 |

| | | | | | | |
|---|---|---|---|---|---|---|
| Q9LV21 | T-complex protein 1 subunit delta OS=Arabidopsis thaliana | 35.63 | 4.66 % | 2 | 57.7 | 7.68 |
| P06525 | Alcohol dehydrogenase class-P OS=Arabidopsis thaliana | 32.46 | 8.71 % | 2 | 41.2 | 6.24 |
| O04450 | T-complex protein 1 subunit epsilon OS=Arabidopsis thaliana | 40.64 | 3.74 % | 2 | 59.3 | 5.66 |
| P17817 | Pyrroline-5-carboxylate reductase OS=Glycine max | 90.53 | 7.30 % | 2 | 28.6 | 8.32 |
| Q9C9K3 | Pyrophosphate--fructose 6-phosphate 1-phosphotransferase subunit alpha 2 OS=Arabidopsis | 62.81 | 3.40 % | 2 | 67.5 | 7.23 |
| P19588 | Lectin DB58 OS=Vigna unguiculata subsp. cylindrica | 99.48 | 6.55 % | 2 | 29.4 | 5.00 |
| Q8SKU2 | Protein TIC 62, chloroplastic OS=Pisum sativum | 55.54 | 4.12 % | 2 | 56.9 | 8.72 |
| Q41607 | Sucrose synthase 2 OS=Tulipa gesneriana | 97.99 | 2.93 % | 2 | 93.0 | 6.38 |
| P85952 | Unknown protein 25 (Fragments) OS=Pseudotsuga menziesii | 50.44 | 59.46 % | 2 | 4.2 | 7.25 |
| P49231 | Profilin-1 OS=Phaseolus vulgaris | 95.99 | 17.56 % | 2 | 14.2 | 5.00 |
| Q03878 | Glycine-rich RNA-binding protein OS=Daucus carota | 139.57 | 15.29 % | 2 | 15.7 | 5.38 |
| O80574 | 4-hydroxy-tetrahydrodipicolinate reductase 1, chloroplastic OS=Arabidopsis thaliana | 138.00 | 7.20 % | 2 | 37.5 | 6.46 |
