# Supplementary material for: Lipoxygenase LOX3 Is the Enigmatic Tocopherol Oxidase in Runner Bean (Phaseolus coccineus)
Source: Antioxidants (Basel). 2024 Feb 29;13(3):301. doi: 10.3390/antiox13030301 (PMC10967512; doi:10.3390/antiox13030301)
Supplement: Supplementary file 1 [file antioxidants-13-00301-s001.zip › Fig. S2 PAGE bands.pdf]

sample	Accession	Description	Score	Coverage	#Peptides	MW[kDa]	calc. pI
1	P27481	Linoleate 9S-lipoxygenase (Fragment) OS=Phaseolus vulgaris	12277.57	78.95 %	61	84.1	6.58
upper band	P38417	Linoleate 9S-lipoxygenase-4 OS=Glycine max	5660.84	34.47 %	26	96.5	6.04
	O23755	Elongation factor 2 OS=Beta vulgaris	2877.44	36.42 %	30	93.7	6.30
	P27480	Linoleate 9S-lipoxygenase 1 OS=Phaseolus vulgaris	2156.96	60.09 %	47	97.1	6.96
	Q01390	Sucrose synthase OS=Vigna radiata var. radiata	2035.67	39.63 %	34	92.0	6.51
	P13708	Sucrose synthase OS=Glycine max	1939.74	35.90 %	30	92.2	6.48
	P27322	Heat shock cognate 70 kDa protein 2 OS=Solanum lycopersicum	1915.88	52.95 %	26	70.7	5.19
	A4GG89	Ribulose bisphosphate carboxylase large chain OS=Phaseolus vulgaris	1899.37	51.26 %	24	52.8	6.39
	Q02909	Phosphoenolpyruvate carboxylase, housekeeping isozyme OS=Glycine max	1778.01	45.50 %	40	110.6	5.96
	P36479	Ribulose bisphosphate carboxylase large chain (Fragment) OS=Asarum canadense	1737.93	39.91 %	19	51.6	6.29
	P25836	Ribulose bisphosphate carboxylase large chain (Fragment) OS=Serenoa repens	1676.43	47.97 %	21	51.8	6.80
	Q32701	Ribulose bisphosphate carboxylase large chain OS=Notothixos subaureus	1578.02	36.42 %	17	52.4	6.68
	P24629	Heat shock cognate 70 kDa protein 1 OS=Solanum lycopersicum	1511.31	37.85 %	18	71.2	5.26
	P22953	Probable mediator of RNA polymerase II transcription subunit 37e OS=Arabidopsis thaliar	1470.21	41.78 %	21	71.3	5.12
	Q33619	Ribulose bisphosphate carboxylase large chain (Fragment) OS=Monarda didyma	1433.62	36.58 %	18	52.4	6.92
	P36484	Ribulose bisphosphate carboxylase large chain (Fragment) OS=Chiococca alba	1317.59	40.55 %	17	52.0	7.02
	Q02735	Phosphoenolpyruvate carboxylase OS=Medicago sativa	1306.17	27.33 %	26	110.8	6.24
	P31926	Sucrose synthase OS=Vicia faba	1259.15	32.88 %	24	92.5	6.15
	Q31750	Ribulose bisphosphate carboxylase large chain (Fragment) OS=Calycophyllum candidissim	1247.87	26.23 %	15	51.9	6.23
	Q33528	Ribulose bisphosphate carboxylase large chain (Fragment) OS=Fleroya rubrostipulata	1240.17	33.26 %	15	52.1	6.68
	P28429	Ribulose bisphosphate carboxylase large chain (Fragment) OS=Lobelia sp.	1237.02	35.19 %	16	52.0	6.93
	P09918	Seed linoleate 9S-lipoxygenase-3 OS=Pisum sativum	1226.31	9.06 %	9	97.6	6.51
	P09439	Seed linoleate 9S-lipoxygenase-2 OS=Glycine max	1090.49	17.34 %	14	97.1	6.74
	P24095	Seed linoleate 9S-lipoxygenase OS=Glycine max	1090.46	19.56 %	18	96.8	6.15
	P51062	Phosphoenolpyruvate carboxylase OS=Pisum sativum	1078.55	27.82 %	26	110.9	6.38
	P22954	Probable mediator of RNA polymerase II transcription subunit 37c OS=Arabidopsis thaliar	1063.19	33.23 %	15	71.3	5.12
	Q9MUT6	Ribulose bisphosphate carboxylase large chain OS=Mesostigma viride	1042.42	20.84 %	11	52.5	6.60
	P09186	Seed linoleate 9S-lipoxygenase-3 OS=Glycine max	983.93	18.55 %	15	96.7	6.73
	Q9C7X7	Heat shock 70 kDa protein 18 OS=Arabidopsis thaliana	980.78	25.77 %	13	68.3	5.35
	P29196	Phosphoenolpyruvate carboxylase OS=Solanum tuberosum	979.91	27.67 %	24	110.2	5.77
	Q69RJ0	Ferredoxin-dependent glutamate synthase, chloroplastic OS=Oryza sativa subsp. japonica	883.23	11.76 %	16	174.9	6.84
	Q9ZNZ7	Ferredoxin-dependent glutamate synthase 1, chloroplastic/mitochondrial OS=Arabidopsis	873.48	11.90 %	15	176.6	6.32
	Q9FGX1	ATP-citrate synthase beta chain protein 2 OS=Arabidopsis thaliana	867.16	39.97 %	19	65.8	7.64
	Q9MAH0	Phosphoenolpyruvate carboxylase 1 OS=Arabidopsis thaliana	845.15	24.20 %	20	110.2	6.06
	P51059	Phosphoenolpyruvate carboxylase 2 OS=Zea mays	834.42	24.20 %	21	109.9	6.04
	P16097	Phosphoenolpyruvate carboxylase 2 OS=Mesembryanthemum crystallinum	764.02	21.35 %	20	109.1	5.86
	P14856	Seed linoleate 9S-lipoxygenase-2 OS=Pisum sativum	732.27	11.46 %	9	97.1	6.54
	P0CG84	Polyubiquitin (Fragment) OS=Nicotiana sylvestris	729.58	70.29 %	6	42.2	7.58
	Q9C522	ATP-citrate synthase beta chain protein 1 OS=Arabidopsis thaliana	728.19	33.88 %	16	65.8	7.81
	Q93VT8	ATP-citrate synthase beta chain protein 1 OS=Oryza sativa subsp. japonica	718.92	29.44 %	15	66.0	7.68
	P19142	Phenylalanine ammonia-lyase class 2 OS=Phaseolus vulgaris	699.38	34.27 %	21	77.3	6.40
	Q32223	Ribulose bisphosphate carboxylase large chain (Fragment) OS=Ephedra tweediana	678.18	23.44 %	13	51.7	6.43
	Q3ZJ74	Ribulose bisphosphate carboxylase large chain OS=Pseudendoclonium akinetum	540.6	19.79 %	8	52.4	6.60
	P08170	Seed linoleate 13S-lipoxygenase-1 OS=Glycine max	533.48	11.20 %	10	94.3	6.39
	Q9T0P4	Ferredoxin-dependent glutamate synthase 2, chloroplastic OS=Arabidopsis thaliana	523.32	7.37 %	10	177.6	7.01
	P49034	Sucrose synthase OS=Alnus glutinosa	515.08	14.57 %	11	91.6	6.81
	Q9FPT1	Ubiquitin carboxyl-terminal hydrolase 12 OS=Arabidopsis thaliana	469.47	9.68 %	13	130.5	5.77
	Q84WU2	Ubiquitin carboxyl-terminal hydrolase 13 OS=Arabidopsis thaliana	463.94	10.04 %	13	130.6	5.58
	P49039	Sucrose synthase OS=Solanum tuberosum	449.66	11.43 %	10	92.5	6.40
	P43210	Delta-aminolevulinic acid dehydratase, chloroplastic OS=Glycine max	433.94	31.31 %	9	45.0	7.09
	O80988	Glycine dehydrogenase (decarboxylating) 2, mitochondrial OS=Arabidopsis thaliana	419.05	10.63 %	9	113.7	6.65
	O82392	Phosphomethylpyrimidine synthase, chloroplastic OS=Arabidopsis thaliana	413.94	17.39 %	10	71.9	6.44
	Q9SN95	UDP-glucuronic acid decarboxylase 5 OS=Arabidopsis thaliana	392.98	31.67 %	8	38.4	7.52
	P27991	Phenylalanine ammonia-lyase 1 OS=Glycine max	381.36	13.18 %	9	77.7	6.42
	Q41140	Pyrophosphate--fructose 6-phosphate 1-phosphotransferase subunit alpha OS=Ricinus cc	323.14	17.18 %	8	67.3	7.62
	O23255	Adenosylhomocysteinase 1 OS=Arabidopsis thaliana	318.86	25.36 %	9	53.3	5.97
	O04379	Protein argonaute 1 OS=Arabidopsis thaliana	300.65	8.49 %	7	116.1	9.29
	P10691	Sucrose synthase OS=Solanum tuberosum	287.9	6.58 %	6	92.4	6.21
	Q8H0S9	Puromycin-sensitive aminopeptidase OS=Arabidopsis thaliana	285.45	10.31 %	11	99.1	5.67
	Q42560	Aconitate hydratase 1 OS=Arabidopsis thaliana	284.42	15.14 %	8	98.1	6.40
	Q41608	Sucrose synthase 1 OS=Tulipa gesneriana	246.39	6.71 %	5	92.2	6.57
	Q7XSA2	Protein argonaute 1B OS=Oryza sativa subsp. japonica	245.66	10.11 %	9	123.5	9.50
	O24534	Elongation factor 1-alpha OS=Vicia faba	239.3	16.33 %	8	49.2	9.07
	Q6K972	Protein argonaute 1C OS=Oryza sativa subsp. japonica	238.7	8.21 %	7	113.1	9.50
	P25698	Elongation factor 1-alpha OS=Glycine max	235.42	12.98 %	7	49.3	9.06
	O49954	Glycine dehydrogenase (decarboxylating), mitochondrial OS=Solanum tuberosum	233.94	9.37 %	7	112.8	6.98
	P50249	Adenosylhomocysteinase OS=Phalaenopsis sp.	229.64	14.43 %	7	53.1	6.14
	P35007	Adenosylhomocysteinase OS=Catharanthus roseus	224.46	16.49 %	7	53.2	5.88
	Q41141	Pyrophosphate--fructose 6-phosphate 1-phosphotransferase subunit beta OS=Ricinus cor	217.61	14.13 %	7	60.1	6.64
	Q9LF33	UDP-glucose 6-dehydrogenase 3 OS=Arabidopsis thaliana	216.9	14.58 %	7	53.1	6.04
	Q9STW6	Heat shock 70 kDa protein 6, chloroplastic OS=Arabidopsis thaliana	213.41	9.05 %	6	76.5	5.20
	Q9XGW1	Protein argonaute 10 OS=Arabidopsis thaliana	211.18	4.45 %	5	110.8	9.23
	Q41607	Sucrose synthase 2 OS=Tulipa gesneriana	211.18	5.61 %	5	93.0	6.38
	P93736	Valine--tRNA ligase OS=Arabidopsis thaliana	211.01	7.13 %	8	125.8	7.11
	Q03460	Glutamate synthase [NADH], amyloplastic OS=Medicago sativa	202.15	4.38 %	9	240.2	6.27

F4JVN6	Tripeptidyl-peptidase 2 OS=Arabidopsis thaliana	196.05	2.54 %	3	152.3	6.07
Q02028	Stromal 70 kDa heat shock-related protein, chloroplastic OS=Pisum sativum	190.44	10.62 %	7	75.5	5.35
Q6ESI7	Tripeptidyl-peptidase 2 OS=Oryza sativa subsp. japonica	190.29	2.87 %	4	148.5	6.58
Q9SGY2	ATP-citrate synthase alpha chain protein 1 OS=Arabidopsis thaliana	177.26	5.67 %	3	46.6	5.50
P52780	Glutamine--tRNA ligase OS=Lupinus luteus	175.34	9.57 %	7	90.4	6.76
F4JLI5	Xanthine dehydrogenase 2 OS=Arabidopsis thaliana	174.28	3.18 %	4	148.7	6.73
P29409	Phosphoglycerate kinase, chloroplastic (Fragment) OS=Spinacia oleracea	172.36	14.55 %	5	45.5	6.16
P23686	S-adenosylmethionine synthase 1 OS=Arabidopsis thaliana	160.61	17.81 %	5	43.1	5.82
Q8W3Y4	S-adenosylmethionine synthase OS=Phaseolus lunatus	154.95	12.24 %	4	43.0	5.94
Q5Z5B2	Protein argonaute 1D OS=Oryza sativa subsp. japonica	154.52	6.17 %	5	115.9	9.03
Q8GSM2	Lipoxygenase 2.3, chloroplastic OS=Hordeum vulgare	150.26	1.90 %	2	101.2	6.29
Q9SF16	T-complex protein 1 subunit eta OS=Arabidopsis thaliana	146.27	9.87 %	5	59.7	6.39
Q96558	UDP-glucose 6-dehydrogenase 1 OS=Glycine max	146.02	7.71 %	4	52.9	6.04
P29185	Chaperonin CPN60-1, mitochondrial OS=Zea mays	141.27	6.07 %	3	61.2	5.85
Q9SFH9	Delta-aminolevulinic acid dehydratase 1, chloroplastic OS=Arabidopsis thaliana	138.86	16.98 %	5	46.7	7.31
P15252	Rubber elongation factor protein OS=Hevea brasiliensis	136.48	46.38 %	4	14.7	5.19
B7EA73	Puromycin-sensitive aminopeptidase OS=Oryza sativa subsp. japonica	135.46	5.64 %	5	99.5	5.59
Q6YZX6	Putative aconitate hydratase, cytoplasmic OS=Oryza sativa subsp. japonica	134.72	5.35 %	4	98.0	6.01
Q43191	Probable linoleate 9S-lipoxygenase 5 OS=Solanum tuberosum	134.16	4.06 %	4	97.7	5.81
P30172	Actin-100 (Fragment) OS=Solanum tuberosum	133.07	18.77 %	4	39.5	5.83
P86387	Phospholipase D alpha 1 OS=Carica papaya	132.39	6.31 %	5	92.0	5.80
Q05045	Chaperonin CPN60-1, mitochondrial OS=Cucurbita maxima	131.72	8.70 %	5	61.0	5.77
P29197	Chaperonin CPN60, mitochondrial OS=Arabidopsis thaliana	127.54	7.80 %	5	61.2	5.78
P53504	Actin-1 OS=Sorghum bicolor	126.23	17.77 %	4	41.8	5.69
P15804	Phosphoenolpyruvate carboxylase 3 OS=Sorghum bicolor	126.09	5.83 %	6	108.3	6.27
P26301	Enolase 1 OS=Zea mays	125.82	12.11 %	5	48.0	5.33
A9PEK8	S-adenosylmethionine synthase 3 OS=Populus trichocarpa	125.77	12.15 %	4	43.1	5.81
Q39659	Glyoxysomal fatty acid beta-oxidation multifunctional protein MFP-a OS=Cucumis sativus	120.08	5.52 %	3	79.1	9.07
O82549	Phospholipase D alpha 1 OS=Brassica oleracea var. capitata	117.66	5.31 %	4	91.8	5.86
Q9SGE0	UDP-D-apiose/UDP-D-xylose synthase 2 OS=Arabidopsis thaliana	117.54	11.05 %	3	43.8	5.87
P21343	Pyrophosphate--fructose 6-phosphate 1-phosphotransferase subunit beta OS=Solanum tu	117.19	9.14 %	5	61.8	6.42
Q9LEI9	Enolase 2 OS=Hevea brasiliensis	117.09	13.48 %	5	47.9	6.29
O81644	Villin-2 OS=Arabidopsis thaliana	115.12	2.36 %	2	107.8	5.29
**Q06327**	**Linoleate 9S-lipoxygenase 1 OS=Arabidopsis thaliana**	**113.63**	**1.86 %**	**2**	**98.0**	**5.52**
Q43321	Enolase OS=Alnus glutinosa	113.33	12.73 %	4	47.6	5.58
Q0DG35	Glutamate synthase 2 [NADH], chloroplastic OS=Oryza sativa subsp. japonica	112.75	2.06 %	4	239.3	7.01
Q2QNG7	ATP-citrate synthase alpha chain protein 3 OS=Oryza sativa subsp. japonica	112.58	4.02 %	2	46.8	5.58
Q9CAG3	Lipoxygenase 6, chloroplastic OS=Arabidopsis thaliana	111.62	2.84 %	2	104.4	7.88
Q9SJK3	Protein argonaute 5 OS=Arabidopsis thaliana	108.9	3.41 %	3	111.0	9.47
O49485	D-3-phosphoglycerate dehydrogenase 1, chloroplastic OS=Arabidopsis thaliana	101.5	6.97 %	4	63.3	6.58
P31300	Cysteine synthase, chloroplastic/chromoplastic OS=Capsicum annuum	100.23	9.09 %	2	40.0	5.27
Q8VZH2	Aminopeptidase M1 OS=Arabidopsis thaliana	100.01	2.28 %	2	98.1	5.50
Q9M8D3	Probable phosphoribosylformylglycinamidine synthase, chloroplastic/mitochondrial OS=A	96.94	3.34 %	3	153.9	5.36
Q53RB0	Probable linoleate 9S-lipoxygenase 4 OS=Oryza sativa subsp. japonica	92.23	3.31 %	3	98.6	7.31
Q94A28	Aconitate hydratase 3, mitochondrial OS=Arabidopsis thaliana	91.99	5.33 %	3	108.4	7.15
P38661	Probable protein disulfide-isomerase A6 OS=Medicago sativa	91.69	6.04 %	2	40.5	5.59
Q43130	Enolase OS=Mesembryanthemum crystallinum	89.43	9.01 %	3	48.3	5.85
**P38414**	**Linoleate 9S-lipoxygenase OS=Lens culinaris**	**86.43**	**2.42 %**	**4**	**96.6**	**6.44**
Q42669	Aconitate hydratase (Fragment) OS=Cucumis melo var. conomon	85.85	2.62 %	2	83.2	6.80
O22718	ATP-citrate synthase alpha chain protein 2 OS=Arabidopsis thaliana	85.33	6.15 %	2	46.7	5.27
P80607	Alpha-1,4-glucan-protein synthase [UDP-forming] OS=Zea mays	84.9	12.91 %	4	41.2	6.13
P38418	Lipoxygenase 2, chloroplastic OS=Arabidopsis thaliana	84.17	2.34 %	2	102.0	5.62
Q9SYP2	Pyrophosphate--fructose 6-phosphate 1-phosphotransferase subunit alpha 1 OS=Arabido	83.72	7.49 %	5	67.1	6.99
Q9AWB2	Sulfite reductase [ferredoxin], chloroplastic (Fragment) OS=Glycine max	81.98	3.66 %	2	63.8	9.06
Q43621	Glutathione reductase, cytosolic OS=Pisum sativum	81.59	5.82 %	2	53.9	7.01
Q9SVN5	Probable methionine--tRNA ligase OS=Arabidopsis thaliana	81.2	4.52 %	3	89.8	7.01
Q53JY8	ATP-citrate synthase subunit alpha chain protein 1 OS=Oryza sativa subsp. japonica	81.07	8.35 %	2	45.0	6.07
Q01289	Protochlorophyllide reductase, chloroplastic OS=Pisum sativum	80.93	9.02 %	3	42.9	9.04
Q9FLW9	Plastidial pyruvate kinase 2 OS=Arabidopsis thaliana	80.4	5.01 %	3	63.5	7.03
Q40466	Eukaryotic initiation factor 4A-13 (Fragment) OS=Nicotiana tabacum	79.75	6.48 %	2	40.2	5.33
P21218	Protochlorophyllide reductase B, chloroplastic OS=Arabidopsis thaliana	78.52	11.22 %	4	43.3	9.16
P27774	Phosphoribulokinase, chloroplastic OS=Mesembryanthemum crystallinum	74.33	12.34 %	5	44.1	6.46
Q42662	5-methyltetrahydropteroyltriglutamate--homocysteine methyltransferase OS=Plectranthu	73.98	4.32 %	3	84.5	6.51
Q9XES0	1-deoxy-D-xylulose 5-phosphate reductoisomerase, chloroplastic OS=Mentha piperita	72.76	5.11 %	2	51.0	6.27
Q2QTC2	Phosphoglucan, water dikinase, chloroplastic OS=Oryza sativa subsp. japonica	72.05	2.07 %	3	132.8	5.78
P56821	Eukaryotic translation initiation factor 3 subunit B OS=Nicotiana tabacum	71.98	4.03 %	3	82.5	5.02
P54765	GTP-binding nuclear protein Ran1A (Fragment) OS=Lotus japonicus	69.18	9.09 %	2	23.8	7.12
Q9ZPS3	Glutamate decarboxylase 4 OS=Arabidopsis thaliana	68.4	6.29 %	2	56.0	6.34
O81154	Cysteine synthase OS=Solanum tuberosum	67.07	7.38 %	2	34.3	6.62
O22518	40S ribosomal protein SA OS=Glycine max	65.01	10.32 %	3	33.9	5.26
Q9LSH2	Glutamate decarboxylase 5 OS=Arabidopsis thaliana	64.78	5.87 %	2	55.7	5.66
Q07176	Mitogen-activated protein kinase homolog MMK1 OS=Medicago sativa	64.59	6.72 %	2	44.4	5.85
Q9M401	Branched-chain-amino-acid aminotransferase 3, chloroplastic OS=Arabidopsis thaliana	62.89	4.60 %	2	44.9	8.10
Q42601	Carbamoyl-phosphate synthase large chain, chloroplastic OS=Arabidopsis thaliana	61.44	2.27 %	3	129.9	5.73
Q6ZY51	Phosphoglucan, water dikinase, chloroplastic OS=Arabidopsis thaliana	61.37	1.25 %	2	131.2	6.24
Q38931	Peptidyl-prolyl cis-trans isomerase FKBP62 OS=Arabidopsis thaliana	51.27	4.36 %	2	61.4	5.31

2	**P27481**	**Linoleate 9S-lipoxygenase (Fragment) OS=Phaseolus vulgaris**	**11605.42**	**79.89 %**	**52**	**84.1**	**6.58**
lower band	**P38417**	**Linoleate 9S-lipoxygenase-4 OS=Glycine max**	**5724.9**	**24.85 %**	**20**	**96.5**	**6.04**
	P27322	Heat shock cognate 70 kDa protein 2 OS=Solanum lycopersicum	4265.64	57.61 %	31	70.7	5.19
	Q01390	Sucrose synthase OS=Vigna radiata var. radiata	3589.49	57.64 %	41	92.0	6.51
	P13708	Sucrose synthase OS=Glycine max	3477.56	51.93 %	38	92.2	6.48
	O23755	Elongation factor 2 OS=Beta vulgaris	3455.85	35.23 %	28	93.7	6.30
	P24629	Heat shock cognate 70 kDa protein 1 OS=Solanum lycopersicum	3161.4	42.31 %	22	71.2	5.26
	P48715	Ribulose bisphosphate carboxylase large chain (Fragment) OS=Sinapis alba	3058.16	54.12 %	25	52.4	6.68
	P29357	Chloroplast envelope membrane 70 kDa heat shock-related protein OS=Spinacia oleracea	2909.07	42.57 %	21	71.7	5.48
	P28442	Ribulose bisphosphate carboxylase large chain (Fragment) OS=Platytheca verticillata	2889.42	50.97 %	22	51.6	6.48
	P22953	Probable mediator of RNA polymerase II transcription subunit 37e OS=Arabidopsis thaliar	2867.85	51.31 %	27	71.3	5.12
	Q32040	Ribulose bisphosphate carboxylase large chain (Fragment) OS=Cornus obliqua	2811.8	49.25 %	22	51.5	6.44
	P69572	Ribulose bisphosphate carboxylase large chain (Fragment) OS=Lupinus atlanticus	2792.16	48.79 %	21	50.3	6.68
	Q9FGX1	ATP-citrate synthase beta chain protein 2 OS=Arabidopsis thaliana	2695.83	45.72 %	24	65.8	7.64
	Q32041	Ribulose bisphosphate carboxylase large chain (Fragment) OS=Cornus oblonga	2667.01	48.39 %	20	51.6	6.58
	P28436	Ribulose bisphosphate carboxylase large chain (Fragment) OS=Oxalis dillenii	2640.81	48.07 %	19	51.7	6.33
	Q31672	Ribulose bisphosphate carboxylase large chain (Fragment) OS=Antirhea lucida	2538.11	40.72 %	19	52.1	6.92
	P28419	Ribulose bisphosphate carboxylase large chain (Fragment) OS=Hamamelis mollis	2397.07	49.25 %	22	51.5	6.48
	Q33528	Ribulose bisphosphate carboxylase large chain (Fragment) OS=Fleroya rubrostipulata	2328.82	46.27 %	17	52.1	6.68
	**P27480**	**Linoleate 9S-lipoxygenase 1 OS=Phaseolus vulgaris**	**2322.8**	**53.13 %**	**36**	**97.1**	**6.96**
	Q93VT8	ATP-citrate synthase beta chain protein 1 OS=Oryza sativa subsp. japonica	2309.83	37.34 %	19	66.0	7.68
	P36484	Ribulose bisphosphate carboxylase large chain (Fragment) OS=Chiococca alba	2295.29	42.68 %	19	52.0	7.02
	Q9C522	ATP-citrate synthase beta chain protein 1 OS=Arabidopsis thaliana	2244.49	43.09 %	22	65.8	7.81
	Q31750	Ribulose bisphosphate carboxylase large chain (Fragment) OS=Calycophyllum candidissim	2179.31	33.69 %	17	51.9	6.23
	Q8W4M5	Pyrophosphate--fructose 6-phosphate 1-phosphotransferase subunit beta 1 OS=Arabidop	2159.12	24.20 %	15	61.4	6.10
	Q33619	Ribulose bisphosphate carboxylase large chain (Fragment) OS=Monarda didyma	2063.08	40.38 %	19	52.4	6.92
	P34767	Ribulose bisphosphate carboxylase large chain (Fragment) OS=Alisma plantago-aquatica	1933.73	47.21 %	18	43.9	7.15
	Q41141	Pyrophosphate--fructose 6-phosphate 1-phosphotransferase subunit beta OS=Ricinus cor	1883.44	23.91 %	17	60.1	6.64
	P19142	Phenylalanine ammonia-lyase class 2 OS=Phaseolus vulgaris	1719.73	60.39 %	30	77.3	6.40
	Q9C7X7	Heat shock 70 kDa protein 18 OS=Arabidopsis thaliana	1656.45	31.60 %	16	68.3	5.35
	P26413	Heat shock 70 kDa protein OS=Glycine max	1580.86	19.69 %	9	70.8	5.49
	Q05046	Chaperonin CPN60-2, mitochondrial OS=Cucurbita maxima	1538.53	33.04 %	19	61.1	6.64
	Q9MUT6	Ribulose bisphosphate carboxylase large chain OS=Mesostigma viride	1514.11	25.05 %	13	52.5	6.60
	Q02909	Phosphoenolpyruvate carboxylase, housekeeping isozyme OS=Glycine max	1423.67	40.54 %	31	110.6	5.96
	Q01899	Heat shock 70 kDa protein, mitochondrial OS=Phaseolus vulgaris	1414.6	45.33 %	23	72.5	6.20
	Q41140	Pyrophosphate--fructose 6-phosphate 1-phosphotransferase subunit alpha OS=Ricinus co	1412.77	26.74 %	13	67.3	7.62
	Q9AWB2	Sulfite reductase [ferredoxin], chloroplastic (Fragment) OS=Glycine max	1339.52	40.49 %	20	63.8	9.06
	P51061	Phosphoenolpyruvate carboxylase OS=Glycine max	1281.18	28.23 %	23	110.7	6.47
	Q05045	Chaperonin CPN60-1, mitochondrial OS=Cucurbita maxima	1249.43	30.09 %	19	61.0	5.77
	Q8L7B5	Chaperonin CPN60-like 1, mitochondrial OS=Arabidopsis thaliana	1217.22	24.27 %	12	61.9	6.68
	P21343	Pyrophosphate--fructose 6-phosphate 1-phosphotransferase subunit beta OS=Solanum tu	1216.15	20.39 %	14	61.8	6.42
	P12466	Ribulose bisphosphate carboxylase large chain OS=Chlorella vulgaris	1208.59	21.68 %	11	52.5	6.48
	P29185	Chaperonin CPN60-1, mitochondrial OS=Zea mays	1193.69	31.54 %	18	61.2	5.85
	Q02028	Stromal 70 kDa heat shock-related protein, chloroplastic OS=Pisum sativum	1192.99	28.33 %	17	75.5	5.35
	O82392	Phosphomethylpyrimidine synthase, chloroplastic OS=Arabidopsis thaliana	1151.11	24.38 %	15	71.9	6.44
	Q9STW6	Heat shock 70 kDa protein 6, chloroplastic OS=Arabidopsis thaliana	1151.09	22.70 %	15	76.5	5.20
	Q96558	UDP-glucose 6-dehydrogenase 1 OS=Glycine max	1144.51	61.46 %	22	52.9	6.04
	Q02735	Phosphoenolpyruvate carboxylase OS=Medicago sativa	1105.38	23.40 %	21	110.8	6.24
	Q42806	Pyruvate kinase, cytosolic isozyme OS=Glycine max	1053.33	47.95 %	20	55.3	7.56
	Q9SIU0	NAD-dependent malic enzyme 1, mitochondrial OS=Arabidopsis thaliana	1052.01	24.40 %	11	69.6	5.45
	P49034	Sucrose synthase OS=Alnus glutinosa	1013.95	16.19 %	12	91.6	6.81
	P29197	Chaperonin CPN60, mitochondrial OS=Arabidopsis thaliana	982.58	23.74 %	16	61.2	5.78
	P24095	Seed linoleate 9S-lipoxygenase OS=Glycine max	967.96	15.97 %	14	96.8	6.15
	P09918	Seed linoleate 9S-lipoxygenase-3 OS=Pisum sativum	962.82	13.59 %	10	97.6	6.51
	Q84WU2	Ubiquitin carboxyl-terminal hydrolase 13 OS=Arabidopsis thaliana	955.77	13.00 %	15	130.6	5.58
	P09439	Seed linoleate 9S-lipoxygenase-2 OS=Glycine max	918.68	15.95 %	13	97.1	6.74
	Q43117	Pyruvate kinase isozyme A, chloroplastic OS=Ricinus communis	864.76	25.56 %	11	64.1	5.57
	Q9FPT1	Ubiquitin carboxyl-terminal hydrolase 12 OS=Arabidopsis thaliana	783.28	11.92 %	14	130.5	5.77
	P49039	Sucrose synthase OS=Solanum tuberosum	781.74	18.26 %	12	92.5	6.40
	P50249	Adenosylhomocysteinase OS=Phalaenopsis sp.	760.11	28.04 %	15	53.1	6.14
	Q9S9N1	Heat shock 70 kDa protein 5 OS=Arabidopsis thaliana	750.65	17.34 %	8	70.9	5.41
	P09186	Seed linoleate 9S-lipoxygenase-3 OS=Glycine max	748.93	12.02 %	11	96.7	6.73
	P07218	Phenylalanine ammonia-lyase class 1 (Fragment) OS=Phaseolus vulgaris	736.64	36.76 %	14	55.8	6.07
	Q39659	Glyoxysomal fatty acid beta-oxidation multifunctional protein MFP-a OS=Cucumis sativus	724.33	8.41 %	5	79.1	9.07
	O23255	Adenosylhomocysteinase 1 OS=Arabidopsis thaliana	723.35	33.61 %	15	53.3	5.97
	P93253	Adenosylhomocysteinase OS=Mesembryanthemum crystallinum	711.51	33.40 %	15	53.1	6.10
	Q9FLW9	Plastidial pyruvate kinase 2 OS=Arabidopsis thaliana	697.68	25.56 %	11	63.5	7.03
	Q43155	Ferredoxin-dependent glutamate synthase, chloroplastic OS=Spinacia oleracea	692.68	10.09 %	13	165.3	6.19
	P45732	Phenylalanine ammonia-lyase OS=Stylosanthes humilis	685.17	22.94 %	12	77.9	6.49
	P27991	Phenylalanine ammonia-lyase 1 OS=Glycine max	664.2	22.44 %	13	77.7	6.42
	P37225	NAD-dependent malic enzyme 59 kDa isoform, mitochondrial OS=Solanum tuberosum	647.68	16.31 %	9	66.2	6.34
	Q9MAH0	Phosphoenolpyruvate carboxylase 1 OS=Arabidopsis thaliana	646.68	17.99 %	14	110.2	6.06
	P49040	Sucrose synthase 1 OS=Arabidopsis thaliana	620.46	19.68 %	10	92.9	6.20
	P21342	Pyrophosphate--fructose 6-phosphate 1-phosphotransferase subunit alpha OS=Solanum t	619.82	13.31 %	6	67.3	7.43
	Q31674	Ribulose bisphosphate carboxylase large chain (Fragment) OS=Anemia mexicana	617.13	20.71 %	9	46.5	7.02

Q9ZNZ7	Ferredoxin-dependent glutamate synthase 1, chloroplastic/mitochondrial OS=Arabidopsis	610.13	8.26 %	10	176.6	6.32
Q75NZ0	Sulfite reductase [ferredoxin], chloroplastic OS=Pisum sativum	606.32	11.68 %	7	76.9	9.03
P52780	Glutamine--tRNA ligase OS=Lupinus luteus	606.25	15.87 %	12	90.4	6.76
Q9LF33	UDP-glucose 6-dehydrogenase 3 OS=Arabidopsis thaliana	549.13	27.92 %	10	53.1	6.04
Q9MAJ7	Beta-galactosidase 5 OS=Arabidopsis thaliana	530.24	8.74 %	5	81.4	8.25
Q41608	Sucrose synthase 1 OS=Tulipa gesneriana	522.52	8.32 %	6	92.2	6.57
P0CG84	Polyubiquitin (Fragment) OS=Nicotiana sylvestris	506.64	62.33 %	4	42.2	7.58
Q08276	Heat shock 70 kDa protein, mitochondrial OS=Solanum tuberosum	496.73	15.40 %	8	73.0	6.76
P43210	Delta-aminolevulinic acid dehydratase, chloroplastic OS=Glycine max	494.22	36.17 %	10	45.0	7.09
P35007	Adenosylhomocysteinase OS=Catharanthus roseus	490.1	20.82 %	12	53.2	5.88
Q9T0P4	Ferredoxin-dependent glutamate synthase 2, chloroplastic OS=Arabidopsis thaliana	475.57	6.08 %	8	177.6	7.01
Q07356	15-cis-phytoene desaturase, chloroplastic/chromoplastic OS=Arabidopsis thaliana	474.18	24.03 %	10	62.9	6.48
P37221	NAD-dependent malic enzyme 62 kDa isoform, mitochondrial OS=Solanum tuberosum	460.33	15.81 %	8	69.9	5.94
Q9SCV9	Beta-galactosidase 3 OS=Arabidopsis thaliana	451.59	10.98 %	8	95.1	7.21
P86387	Phospholipase D alpha 1 OS=Carica papaya	446.74	10.64 %	8	92.0	5.80
Q9SD39	Probable methyltransferase PMT27 OS=Arabidopsis thaliana	445.89	7.82 %	6	101.4	5.52
Q9M568	Phenylalanine ammonia-lyase 1 OS=Rubus idaeus	440.3	11.69 %	8	77.7	6.67
Q43621	Glutathione reductase, cytosolic OS=Pisum sativum	430.3	11.45 %	4	53.9	7.01
P26969	Glycine dehydrogenase (decarboxylating), mitochondrial OS=Pisum sativum	424.52	15.89 %	11	114.6	7.50
Q94IR2	Carotenoid 9,10(9',10')-cleavage dioxygenase 1 OS=Phaseolus vulgaris	424.4	25.41 %	10	61.1	6.54
P10691	Sucrose synthase OS=Solanum tuberosum	421.76	8.20 %	7	92.4	6.21
P93736	Valine--tRNA ligase OS=Arabidopsis thaliana	420.95	5.87 %	8	125.8	7.11
P25872	Phenylalanine ammonia-lyase OS=Nicotiana tabacum	414	17.76 %	9	77.7	6.77
P29828	Protein disulfide-isomerase OS=Medicago sativa	404.42	24.41 %	9	57.1	5.10
Q9LIA8	UDP-glucose 6-dehydrogenase 2 OS=Arabidopsis thaliana	402.31	28.33 %	10	53.1	6.00
Q40545	Pyruvate kinase isozyme A, chloroplastic OS=Nicotiana tabacum	401.99	10.12 %	6	65.2	5.39
O82802	Sulfite reductase 1 [ferredoxin], chloroplastic OS=Nicotiana tabacum	399.51	11.69 %	9	77.9	9.04
Q42560	Aconitate hydratase 1 OS=Arabidopsis thaliana	391.58	16.04 %	9	98.1	6.40
Q9LIK0	Plastidial pyruvate kinase 1, chloroplastic OS=Arabidopsis thaliana	391.04	13.09 %	6	65.1	5.92
P0DH99	Elongation factor 1-alpha 1 OS=Arabidopsis thaliana	385.86	19.38 %	8	49.5	9.11
P28553	Phytoene dehydrogenase, chloroplastic/chromoplastic OS=Glycine max	381.89	20.53 %	9	63.6	7.43
Q8W0A1	Beta-galactosidase 2 OS=Oryza sativa subsp. japonica	374.46	10.76 %	7	91.5	5.90
Q41607	Sucrose synthase 2 OS=Tulipa gesneriana	370.63	7.20 %	6	93.0	6.38
P08824	RuBisCO large subunit-binding protein subunit alpha (Fragment) OS=Ricinus communis	363.67	12.32 %	5	52.3	4.87
P25698	Elongation factor 1-alpha OS=Glycine max	349.43	17.67 %	7	49.3	9.06
P93400	Phospholipase D alpha 1 OS=Nicotiana tabacum	347.56	7.18 %	5	91.9	5.63
P12628	NADP-dependent malic enzyme OS=Phaseolus vulgaris	339.34	20.03 %	11	64.9	6.86
O04379	Protein argonaute 1 OS=Arabidopsis thaliana	336.36	8.02 %	7	116.1	9.29
Q9SF16	T-complex protein 1 subunit eta OS=Arabidopsis thaliana	330.72	16.16 %	9	59.7	6.39
Q9LEI9	Enolase 2 OS=Hevea brasiliensis	326.03	17.75 %	6	47.9	6.29
O49954	Glycine dehydrogenase (decarboxylating), mitochondrial OS=Solanum tuberosum	317.02	11.88 %	9	112.8	6.98
Q6K669	Leucine aminopeptidase 2, chloroplastic OS=Oryza sativa subsp. japonica	303.63	11.54 %	6	61.8	8.10
O82549	Phospholipase D alpha 1 OS=Brassica oleracea var. capitata	301.42	8.52 %	7	91.8	5.86
P42896	Enolase OS=Ricinus communis	299.69	17.98 %	6	47.9	5.78
Q40079	V-type proton ATPase subunit B 2 OS=Hordeum vulgare	297.68	18.84 %	8	53.7	5.24
O82191	NADP-dependent malic enzyme 1 OS=Arabidopsis thaliana	291.53	16.18 %	8	64.2	6.76
P21239	RuBisCO large subunit-binding protein subunit alpha, chloroplastic (Fragment) OS=Brassic	289	13.00 %	4	57.7	4.93
Q7XSA2	Protein argonaute 1B OS=Oryza sativa subsp. japonica	287.33	9.57 %	8	123.5	9.50
Q39256	Polyubiquitin 8 OS=Arabidopsis thaliana	284.72	9.19 %	4	71.7	9.03
P08170	Seed linoleate 13S-lipoxygenase-1 OS=Glycine max	283.27	9.42 %	7	94.3	6.39
Q9FL12	Protease Do-like 9 OS=Arabidopsis thaliana	280.22	9.80 %	5	65.1	6.81
P29409	Phosphoglycerate kinase, chloroplastic (Fragment) OS=Spinacia oleracea	279.64	26.10 %	8	45.5	6.16
P51615	NADP-dependent malic enzyme OS=Vitis vinifera	279.33	17.43 %	9	65.2	6.49
Q9LV33	Beta-glucosidase 44 OS=Arabidopsis thaliana	278.46	8.40 %	5	58.9	9.42
Q8L7V3	Probable methyltransferase PMT26 OS=Arabidopsis thaliana	275.02	7.84 %	6	93.0	5.16
Q9C9K3	Pyrophosphate--fructose 6-phosphate 1-phosphotransferase subunit alpha 2 OS=Arabido	274.65	11.18 %	5	67.5	7.23
P19143	Phenylalanine ammonia-lyase class 3 OS=Phaseolus vulgaris	274.48	12.39 %	7	77.9	5.72
Q8RU27	Alpha-1,4-glucan-protein synthase [UDP-forming] 2 OS=Solanum tuberosum	273	21.58 %	6	41.6	6.07
Q9LEJ0	Enolase 1 OS=Hevea brasiliensis	270.33	16.40 %	5	47.8	5.80
Q38931	Peptidyl-prolyl cis-trans isomerase FKBP62 OS=Arabidopsis thaliana	268.34	8.17 %	6	61.4	5.31
Q0WT31	Probable methyltransferase PMT25 OS=Arabidopsis thaliana	266.75	6.36 %	5	86.8	5.40
O80988	Glycine dehydrogenase (decarboxylating) 2, mitochondrial OS=Arabidopsis thaliana	265.7	10.44 %	8	113.7	6.65
Q43130	Enolase OS=Mesembryanthemum crystallinum	262.15	9.68 %	5	48.3	5.85
Q9SS45	Phenylalanine ammonia-lyase 4 OS=Arabidopsis thaliana	256.35	14.71 %	8	76.9	6.27
Q42962	Phosphoglycerate kinase, cytosolic OS=Nicotiana tabacum	256.16	20.45 %	7	42.3	5.97
P42066	Phosphoenolpyruvate carboxykinase [ATP] OS=Cucumis sativus	255.97	10.45 %	6	74.4	6.54
O49485	D-3-phosphoglycerate dehydrogenase 1, chloroplastic OS=Arabidopsis thaliana	254.55	10.12 %	6	63.3	6.58
P34105	NADP-dependent malic enzyme OS=Populus trichocarpa	252.52	17.60 %	8	65.2	6.96
Q9XGW1	Protein argonaute 10 OS=Arabidopsis thaliana	244.02	5.77 %	5	110.8	9.23
Q9SGY2	ATP-citrate synthase alpha chain protein 1 OS=Arabidopsis thaliana	243.14	9.69 %	5	46.6	5.50
Q8L7K9	NAD-dependent malic enzyme 2, mitochondrial OS=Arabidopsis thaliana	242.63	11.86 %	9	66.6	7.06
Q43321	Enolase OS=Alnus glutinosa	239.37	14.77 %	4	47.6	5.58
Q9SN95	UDP-glucuronic acid decarboxylase 5 OS=Arabidopsis thaliana	231.7	17.89 %	6	38.4	7.52
Q42919	Glucose-6-phosphate 1-dehydrogenase, cytoplasmic isoform OS=Medicago sativa	229.64	17.86 %	7	58.9	6.21
Q40024	Betaine aldehyde dehydrogenase OS=Hordeum vulgare	226.47	9.31 %	3	54.3	6.18
Q84XU2	Serine/threonine-protein phosphatase 5 OS=Arabidopsis thaliana	226.4	9.85 %	4	60.2	6.84

Q9S7C0	Heat shock 70 kDa protein 14 OS=Arabidopsis thaliana	226.3	4.33 %	4	91.7	5.25
Q40546	Pyruvate kinase isozyme G, chloroplastic OS=Nicotiana tabacum	225.44	9.25 %	6	61.8	7.53
P55964	Pyruvate kinase isozyme G, chloroplastic (Fragment) OS=Ricinus communis	221.3	8.37 %	3	46.0	6.58
Q6ESI7	Tripeptidyl-peptidase 2 OS=Oryza sativa subsp. japonica	220.94	3.31 %	5	148.5	6.58
Q8VZC3	Delta-1-pyrroline-5-carboxylate dehydrogenase 12A1, mitochondrial OS=Arabidopsis thal	219.92	9.89 %	6	61.7	6.73
P34094	Phytochrome B OS=Solanum tuberosum	219.12	4.25 %	4	125.3	6.04
Q9T074	Phosphoenolpyruvate carboxykinase [ATP] OS=Arabidopsis thaliana	216.81	8.05 %	4	73.4	7.08
Q944P7	Leucine aminopeptidase 3, chloroplastic OS=Arabidopsis thaliana	215.47	9.09 %	5	61.3	7.08
Q9SFH9	Delta-aminolevulinic acid dehydratase 1, chloroplastic OS=Arabidopsis thaliana	213.64	16.98 %	5	46.7	7.31
Q9SLZ0	Phosphoenolpyruvate carboxykinase [ATP] OS=Zea mays	213.62	10.66 %	5	73.3	7.03
O04883	Phospholipase D alpha 1 OS=Pimpinella brachycarpa	211.09	3.96 %	4	91.6	6.11
Q43207	70 kDa peptidyl-prolyl isomerase OS=Triticum aestivum	203.9	6.62 %	5	62.0	5.40
O82261	Protease Do-like 2, chloroplastic OS=Arabidopsis thaliana	202.93	6.26 %	3	66.8	6.05
Q93ZM7	Chaperonin CPN60-like 2, mitochondrial OS=Arabidopsis thaliana	202.38	7.17 %	3	60.4	6.10
P00865	Ribulose bisphosphate carboxylase small chain 1, chloroplastic OS=Glycine max	199.02	13.48 %	3	20.1	8.66
P51850	Pyruvate decarboxylase 1 OS=Pisum sativum	197.74	6.91 %	4	63.9	5.72
F4JVN6	Tripeptidyl-peptidase 2 OS=Arabidopsis thaliana	196.55	3.41 %	4	152.3	6.07
B3VMC0	Betaine aldehyde dehydrogenase 2 OS=Oryza sativa subsp. indica	196.25	8.95 %	3	54.6	5.45
Q9ZPI5	Peroxisomal fatty acid beta-oxidation multifunctional protein MFP2 OS=Arabidopsis thali	195.16	4.69 %	4	78.8	9.17
Q9M8D3	Probable phosphoribosylformylglycinamidine synthase, chloroplastic/mitochondrial OS=A	193.68	2.99 %	3	153.9	5.36
Q5QMT0	Beta-glucosidase 1 OS=Oryza sativa subsp. japonica	193.09	5.04 %	4	58.0	8.27
O24076	Guanine nucleotide-binding protein subunit beta-like protein OS=Medicago sativa	192.94	16.00 %	5	35.6	7.44
Q9FFT4	Pyruvate decarboxylase 2 OS=Arabidopsis thaliana	192.15	6.59 %	4	65.8	5.96
P42499	Phytochrome B OS=Glycine max	191.39	5.54 %	5	129.0	6.28
Q9AXQ8	Deoxyhypusine synthase OS=Dianthus caryophyllus	191.24	12.87 %	4	41.1	5.83
F4JLI5	Xanthine dehydrogenase 2 OS=Arabidopsis thaliana	191.03	3.47 %	4	148.7	6.73
P55826	Protoporphyrinogen oxidase 1, chloroplastic OS=Arabidopsis thaliana	190.58	8.19 %	3	57.7	9.03
P43279	NADP-dependent malic enzyme, chloroplastic OS=Oryza sativa subsp. japonica	189.23	10.95 %	5	69.8	7.14
Q9LV34	Beta-glucosidase 43 OS=Arabidopsis thaliana	188.22	5.19 %	4	57.2	7.25
P31300	Cysteine synthase, chloroplastic/chromoplastic OS=Capsicum annuum	188.08	12.57 %	3	40.0	5.27
Q06060	Mitogen-activated protein kinase homolog D5 OS=Pisum sativum	186.42	17.77 %	4	45.1	5.73
Q9CAG3	Lipoxygenase 6, chloroplastic OS=Arabidopsis thaliana	184.49	4.91 %	3	104.4	7.88
Q8H0S9	Puromycin-sensitive aminopeptidase OS=Arabidopsis thaliana	182.53	6.12 %	5	99.1	5.67
P02581	Actin-1 OS=Glycine max	178.66	14.32 %	3	41.3	5.58
P08927	RuBisCO large subunit-binding protein subunit beta, chloroplastic OS=Pisum sativum	177.79	6.89 %	4	62.9	6.04
P54765	GTP-binding nuclear protein Ran1A (Fragment) OS=Lotus japonicus	176.47	28.23 %	6	23.8	7.12
P37224	NAD-dependent malic enzyme 65 kDa isoform, mitochondrial OS=Amaranthus hypochon	174.57	9.47 %	4	69.6	5.91
Q43191	Probable linoleate 9S-lipoxygenase 5 OS=Solanum tuberosum	170.75	5.22 %	5	97.7	5.81
Q0JLS6	Protein ROOT HAIR DEFECTIVE 3 OS=Oryza sativa subsp. japonica	169.41	4.71 %	5	90.4	6.15
Q9LSH2	Glutamate decarboxylase 5 OS=Arabidopsis thaliana	168.69	10.12 %	4	55.7	5.66
P55195	Phosphoribosylaminoimidazole carboxylase, chloroplastic (Fragment) OS=Vigna aconitifol	167.52	7.72 %	3	60.9	6.42
O22607	WD-40 repeat-containing protein MSI4 OS=Arabidopsis thaliana	166.73	14.99 %	4	55.7	6.23
Q43468	Hsp70-Hsp90 organizing protein 1 OS=Glycine max	162.95	6.99 %	3	64.1	5.69
**O24370**	**Linoleate 13S-lipoxygenase 2-1, chloroplastic OS=Solanum tuberosum**	**161.16**	**2.89 %**	**3**	**101.9**	**6.55**
Q9LJE4	Chaperonin 60 subunit beta 2, chloroplastic OS=Arabidopsis thaliana	160.17	7.21 %	4	63.3	5.73
Q39836	Guanine nucleotide-binding protein subunit beta-like protein OS=Glycine max	157.57	13.85 %	5	35.6	7.72
Q339X2	Beta-glucosidase 34 OS=Oryza sativa subsp. japonica	156.76	6.08 %	3	58.0	6.67
Q940P8	T-complex protein 1 subunit beta OS=Arabidopsis thaliana	156.26	11.01 %	4	57.2	5.87
P24007	Ribulose bisphosphate carboxylase small chain, chloroplastic OS=Pyrus pyrifolia	153.84	14.75 %	3	20.5	8.87
P26301	Enolase 1 OS=Zea mays	149.43	14.80 %	6	48.0	5.33
Q42521	Glutamate decarboxylase 1 OS=Arabidopsis thaliana	149.41	15.34 %	4	57.0	5.53
Q6Z382	Coatomer subunit gamma-2 OS=Oryza sativa subsp. japonica	149.12	3.71 %	2	98.5	5.11
Q6RJS2	Deoxyhypusine synthase OS=Brassica napus	148.83	13.86 %	5	41.1	5.50
Q6K972	Protein argonaute 1C OS=Oryza sativa subsp. japonica	148.37	3.56 %	3	113.1	9.50
Q6YZX6	Putative aconitate hydratase, cytoplasmic OS=Oryza sativa subsp. japonica	147.95	6.24 %	5	98.0	6.01
P84577	Putative heat shock protein HSP90 (Fragments) OS=Populus euphratica	146.25	59.57 %	2	5.1	4.03
Q69Q02	4-alpha-glucanotransferase DPE2 OS=Oryza sativa subsp. japonica	146.22	3.28 %	3	108.2	6.40
Q43154	Glutathione reductase, chloroplastic (Fragment) OS=Spinacia oleracea	144.65	9.61 %	4	52.6	5.72
P26302	Phosphoribulokinase, chloroplastic OS=Triticum aestivum	143.63	13.12 %	3	45.1	6.05
Q6ZDY8	Succinate dehydrogenase [ubiquinone] flavoprotein subunit, mitochondrial OS=Oryza sat	143.54	6.03 %	3	68.8	7.08
P93028	Ubiquitin-activating enzyme E1 1 OS=Arabidopsis thaliana	143.29	5.46 %	5	120.2	5.27
Q9CA83	NADP-dependent malic enzyme 4, chloroplastic OS=Arabidopsis thaliana	140.8	11.46 %	5	71.1	6.58
Q9SGE0	UDP-D-apiose/UDP-D-xylose synthase 2 OS=Arabidopsis thaliana	139.63	16.71 %	5	43.8	5.87
P36875	Protein phosphatase PP2A regulatory subunit A (Fragment) OS=Pisum sativum	139.55	16.96 %	4	43.9	5.17
Q69VD5	Protein argonaute PNH1 OS=Oryza sativa subsp. japonica	137.98	4.80 %	3	108.3	9.20
P52588	Protein disulfide-isomerase OS=Zea mays	137.39	6.82 %	4	57.1	5.41
P25697	Phosphoribulokinase, chloroplastic OS=Arabidopsis thaliana	137.26	11.90 %	3	44.4	6.05
Q94K05	T-complex protein 1 subunit theta OS=Arabidopsis thaliana	137.03	6.56 %	3	58.9	5.35
Q9SU63	Aldehyde dehydrogenase family 2 member B4, mitochondrial OS=Arabidopsis thaliana	135.06	6.13 %	3	58.6	7.46
Q42536	Protochlorophyllide reductase A, chloroplastic OS=Arabidopsis thaliana	134.89	10.12 %	4	43.8	9.36
P92974	Ubiquitin-activating enzyme E1 2 OS=Arabidopsis thaliana	133.2	4.92 %	5	119.5	5.29
Q53JY8	ATP-citrate synthase subunit alpha chain protein 1 OS=Oryza sativa subsp. japonica	132.45	12.53 %	4	45.0	6.07
B9SQI7	Probable bifunctional methylthioribulose-1-phosphate dehydratase/enolase-phosphatase	130.87	4.17 %	2	57.7	6.00
P17202	Betaine aldehyde dehydrogenase, chloroplastic OS=Spinacia oleracea	128.91	4.63 %	2	54.2	5.41
P51846	Pyruvate decarboxylase 2 OS=Nicotiana tabacum	128.21	5.54 %	3	67.0	5.96
Q53RB0	Probable linoleate 9S-lipoxygenase 4 OS=Oryza sativa subsp. japonica	127.7	4.45 %	4	98.6	7.31

Q84WV1	T-complex protein 1 subunit gamma OS=Arabidopsis thaliana	126.72	5.95 %	3	60.3	5.77
Q93VK5	Protein LUTEIN DEFICIENT 5, chloroplastic OS=Arabidopsis thaliana	125.6	4.54 %	2	66.8	6.13
Q9XF61	Protein disulfide-isomerase OS=Datisca glomerata	125.08	6.11 %	2	57.1	4.93
Q41638	Xyloglucan endotransglucosylase/hydrolase protein A OS=Phaseolus angularis	123.95	10.27 %	2	33.9	7.42
Q03460	Glutamate synthase [NADH], amyloplastic OS=Medicago sativa	121.76	1.91 %	4	240.2	6.27
Q2QLY4	5-methyltetrahydropteroyltriglutamate--homocysteine methyltransferase 2 OS=Oryza sat	120.22	5.35 %	4	84.6	6.30
Q94A28	Aconitate hydratase 3, mitochondrial OS=Arabidopsis thaliana	119.82	4.92 %	4	108.4	7.15
Q9M0S5	Isoamylase 3, chloroplastic OS=Arabidopsis thaliana	119.74	2.88 %	2	86.3	6.05
Q9FGY1	Beta-D-xylosidase 1 OS=Arabidopsis thaliana	118.35	2.71 %	2	83.5	8.51
O22518	40S ribosomal protein SA OS=Glycine max	117.82	10.32 %	3	33.9	5.26
A2XAZ3	Alcohol dehydrogenase class-3 OS=Oryza sativa subsp. indica	116.37	7.61 %	2	40.8	7.17
Q9SVN5	Probable methionine--tRNA ligase OS=Arabidopsis thaliana	115.39	4.52 %	4	89.8	7.01
Q93Z53	Plastidial pyruvate kinase 3, chloroplastic OS=Arabidopsis thaliana	115.05	7.18 %	4	62.6	7.81
Q96482	Actin-41 (Fragment) OS=Solanum lycopersicum	113.77	11.90 %	2	37.1	5.69
Q9S795	Betaine aldehyde dehydrogenase 1, chloroplastic OS=Arabidopsis thaliana	113.44	4.99 %	2	54.4	5.29
Q9SMZ4	Alpha-aminoadipic semialdehyde synthase OS=Arabidopsis thaliana	113.37	2.63 %	3	117.1	6.05
F4JJJ3	External alternative NAD(P)H-ubiquinone oxidoreductase B3, mitochondrial OS=Arabidop	113.11	6.72 %	3	65.1	8.35
Q9C7N2	MACPF domain-containing protein CAD1 OS=Arabidopsis thaliana	112.91	4.46 %	2	62.2	8.05
Q9LV21	T-complex protein 1 subunit delta OS=Arabidopsis thaliana	112.16	7.46 %	3	57.7	7.68
P37830	Glucose-6-phosphate 1-dehydrogenase, cytoplasmic isoform OS=Solanum tuberosum	111.74	5.48 %	3	58.4	6.34
P15252	Rubber elongation factor protein OS=Hevea brasiliensis	111.51	31.88 %	3	14.7	5.19
P17562	S-adenosylmethionine synthase 2 OS=Arabidopsis thaliana	109.9	13.23 %	3	43.2	6.06
O04130	D-3-phosphoglycerate dehydrogenase 2, chloroplastic OS=Arabidopsis thaliana	105.89	4.65 %	3	66.4	6.13
P38661	Probable protein disulfide-isomerase A6 OS=Medicago sativa	105.52	6.04 %	2	40.5	5.59
Q9C550	2-isopropylmalate synthase 2, chloroplastic OS=Arabidopsis thaliana	105.04	6.02 %	2	68.1	6.37
Q9FKE9	Protein ROOT HAIR DEFECTIVE 3 homolog 2 OS=Arabidopsis thaliana	104.57	4.08 %	4	93.5	6.29
Q43011	Asparagine synthetase [glutamine-hydrolyzing] 2 OS=Oryza sativa subsp. japonica	100.69	4.57 %	2	66.2	6.46
P20973	Ubiquitin-activating enzyme E1 1 OS=Triticum aestivum	100.29	4.19 %	4	116.9	5.31
P31252	Ubiquitin-activating enzyme E1 3 OS=Triticum aestivum	99.95	5.13 %	4	116.4	5.55
Q9LF41	Probable ubiquitin conjugation factor E4 OS=Arabidopsis thaliana	99.79	2.99 %	3	117.5	5.62
Q9LPW7	Protein AUXIN SIGNALING F-BOX 3 OS=Arabidopsis thaliana	99.14	7.28 %	4	64.9	7.21
**Q9LUW0**	**Linoleate 9S-lipoxygenase 5, chloroplastic OS=Arabidopsis thaliana**	**98.6**	**4.63 %**	**4**	**101.0**	**6.62**
O64879	Beta-glucosidase 15 OS=Arabidopsis thaliana	98.08	3.16 %	2	56.9	7.71
Q41249	Protochlorophyllide reductase, chloroplastic OS=Cucumis sativus	98.06	8.54 %	3	43.0	8.91
Q06801	4-alpha-glucanotransferase, chloroplastic/amyloplastic OS=Solanum tuberosum	96.84	4.86 %	2	64.9	5.48
Q43593	Acyl-[acyl-carrier-protein] desaturase, chloroplastic OS=Olea europaea	95.34	5.90 %	2	44.6	6.55
P38418	Lipoxygenase 2, chloroplastic OS=Arabidopsis thaliana	94.75	2.34 %	2	102.0	5.62
O81645	Villin-3 OS=Arabidopsis thaliana	94.38	2.07 %	2	106.3	5.85
Q9FZE0	Beta-glucosidase 40 OS=Arabidopsis thaliana	93.99	4.71 %	3	58.1	8.02
Q9SJK3	Protein argonaute 5 OS=Arabidopsis thaliana	93.56	2.61 %	2	111.0	9.47
**P38414**	**Linoleate 9S-lipoxygenase OS=Lens culinaris**	**91.92**	**2.89 %**	**3**	**96.6**	**6.44**
Q9LFU1	Asparagine synthetase [glutamine-hydrolyzing] 3 OS=Arabidopsis thaliana	91.4	4.67 %	2	65.2	6.57
O48921	Cytochrome P450 97B2, chloroplastic OS=Glycine max	91.33	5.21 %	2	64.7	6.96
Q9FJI5	Glucose-6-phosphate 1-dehydrogenase, cytoplasmic isoform 2 OS=Arabidopsis thaliana	90.64	9.71 %	5	59.1	6.43
Q9LIR4	Dihydroxy-acid dehydratase, chloroplastic OS=Arabidopsis thaliana	90.43	6.25 %	3	64.9	6.23
Q9SGZ5	Probable beta-D-xylosidase 7 OS=Arabidopsis thaliana	90.3	3.52 %	3	83.8	8.03
Q8GXV5	Ureidoglycine aminohydrolase OS=Arabidopsis thaliana	88.25	8.05 %	2	33.7	6.38
Q40466	Eukaryotic initiation factor 4A-13 (Fragment) OS=Nicotiana tabacum	87.28	10.70 %	4	40.2	5.33
O23735	Cysteine synthase OS=Brassica juncea	85.31	9.88 %	3	34.1	5.59
Q9LK23	Glucose-6-phosphate 1-dehydrogenase, cytoplasmic isoform 1 OS=Arabidopsis thaliana	82.98	8.33 %	5	59.1	8.31
O04450	T-complex protein 1 subunit epsilon OS=Arabidopsis thaliana	82.91	3.74 %	2	59.3	5.66
Q9M291	Protein transport protein Sec24-like CEF OS=Arabidopsis thaliana	81.81	1.64 %	2	117.6	7.21
O81154	Cysteine synthase OS=Solanum tuberosum	77.89	7.38 %	2	34.3	6.62
P21218	Protochlorophyllide reductase B, chloroplastic OS=Arabidopsis thaliana	76.62	6.48 %	3	43.3	9.16
Q0WVL7	Golgin candidate 5 OS=Arabidopsis thaliana	76.58	2.09 %	2	108.3	4.83
P23686	S-adenosylmethionine synthase 1 OS=Arabidopsis thaliana	75.13	15.01 %	4	43.1	5.82
O64765	UDP-N-acetylglucosamine diphosphorylase 2 OS=Arabidopsis thaliana	74.74	4.38 %	2	55.7	5.91
O04916	Aconitate hydratase, cytoplasmic (Fragment) OS=Solanum tuberosum	74.45	10.55 %	3	67.1	6.44
Q9LW29	Protein AUXIN SIGNALING F-BOX 2 OS=Arabidopsis thaliana	74	4.17 %	3	64.6	7.37
Q00834	Cysteine synthase OS=Spinacia oleracea	72.57	7.69 %	2	34.2	5.62
Q2QTC2	Phosphoglucan, water dikinase, chloroplastic OS=Oryza sativa subsp. japonica	72.54	2.07 %	3	132.8	5.78
A9RBS1	Probable bifunctional methylthioribulose-1-phosphate dehydratase/enolase-phosphatase	72.11	4.55 %	3	55.4	6.49
O22907	DEAD-box ATP-dependent RNA helicase 24 OS=Arabidopsis thaliana	71.45	2.50 %	2	83.0	5.88
Q42601	Carbamoyl-phosphate synthase large chain, chloroplastic OS=Arabidopsis thaliana	69.02	2.70 %	3	129.9	5.73
P42500	Phytochrome A OS=Glycine max	66.92	2.56 %	2	125.7	6.61
P56821	Eukaryotic translation initiation factor 3 subunit B OS=Nicotiana tabacum	66.81	4.03 %	3	82.5	5.02
M1BYJ7	External alternative NAD(P)H-ubiquinone oxidoreductase B1, mitochondrial OS=Solanum	66	4.68 %	2	65.1	6.49
Q6ZY51	Phosphoglucan, water dikinase, chloroplastic OS=Arabidopsis thaliana	65.44	1.25 %	2	131.2	6.24
Q38893	Zeta-carotene desaturase, chloroplastic/chromoplastic OS=Arabidopsis thaliana	65.25	3.76 %	2	61.6	7.37
O23627	Glycine--tRNA ligase 1, mitochondrial OS=Arabidopsis thaliana	63.85	3.16 %	3	81.9	7.01
O49901	Zeta-carotene desaturase, chloroplastic/chromoplastic OS=Narcissus pseudonarcissus	63.11	3.83 %	2	63.5	7.68
Q9SDG8	Protein argonaute 4A OS=Oryza sativa subsp. japonica	62.06	2.10 %	2	100.6	8.97
Q8VZC0	UDP-glucuronic acid decarboxylase 1 OS=Arabidopsis thaliana	61.95	5.06 %	2	48.6	9.16
Q9M401	Branched-chain-amino-acid aminotransferase 3, chloroplastic OS=Arabidopsis thaliana	61.12	6.30 %	3	44.9	8.10
P30792	2,3-bisphosphoglycerate-independent phosphoglycerate mutase OS=Zea mays	59.86	3.04 %	2	60.6	5.53
O64882	Beta-glucosidase 17 OS=Arabidopsis thaliana	56.82	2.90 %	2	59.1	5.85

P40115	Cryptochrome-1 OS=Sinapis alba	55.7	5.19 %	3	57.0	7.49
O23813	Sulfite reductase [ferredoxin], chloroplastic OS=Zea mays	53.86	1.57 %	2	70.0	8.69
Q9ZTR1	Spermidine synthase 1 OS=Pisum sativum	52.5	7.78 %	2	36.7	5.11
Q10D00	ATP-dependent RNA helicase SUV3, mitochondrial OS=Oryza sativa subsp. japonica	52.34	2.76 %	2	65.1	8.19
P04715	Ribulose bisphosphate carboxylase small chain SSU11A, chloroplastic OS=Petunia hybrida	51.98	11.67 %	2	20.3	8.12
Q9SNY3	GDP-mannose 4,6 dehydratase 1 OS=Arabidopsis thaliana	51.72	4.43 %	2	40.8	7.11
