# Supplementary material for: Lipoxygenase LOX3 Is the Enigmatic Tocopherol Oxidase in Runner Bean (Phaseolus coccineus)
Source: Antioxidants (Basel). 2024 Feb 29;13(3):301. doi: 10.3390/antiox13030301 (PMC10967512; doi:10.3390/antiox13030301)
Supplement: Supplementary file 1 [file antioxidants-13-00301-s001.zip › Fig. S3 alignment.pdf]

```
LOX6_ABM88259      MPVKQIHGSNSGASCLIPQRPCLRGIPHPSFQLWSRPSSFPSQRKLKRVSHGCRNDVTKI     60
LOX5_AAG42354      ---MFQVPG-------------VS-------GILNPGGHGHNKIKGTVV--LMRKNVLDF     35
LOX3_P27481        ------------------------------------------------------------     0
LOX4_AAF15296      --------M-------------LG-------GIFG---NKGQKIKGNLV--LMRKNVLDI     27
LOX1_P27480        ---------------------------------MFGILNRGHKIKGTVV--LMTKNVFDF     25
LOX2_O24320        -------------------------------MFGILGGGKGHKIKGTVV--LMTKNVLDF     27

LOX6_ABM88259      KAVAAKTAEKKLVKVKAIISVQPTVGGIFSERALERGLDDIKDLLGQSLLLELVSAELDP     120
LOX5_AAG42354      NSVPDLT-------KGNVGGVIGTGL-----GVVGSTVDTLTAFLGRSVSLQLISATQSD     83
LOX3_P27481        ------------------------------------------------------------     0
LOX4_AAF15296      NSIT------------NPANVVDTAF-----DIFGSAIDTVTAFAA-SISVQLISSTKTD     69
LOX1_P27480        NEFVSTT-------RGGIVGAAGGLFGAAT-DIVGGIVDGATAIFSRNIAIQLISATKTD     77
LOX2_O24320        NEVVSTA-------GGGVLGIAGGIFGTAN-KVVGGIVDGATAIFSRNIALQLVSATKTD     79

LOX6_ABM88259      KTQLEKKTLEHFAHRTHQ-----SSKEIRYEAEFEVPESFGEVGALLVENEHRREMFVRE     175
LOX5_AAG42354      ENGKGKVGKDTFIEGIITSLPTLGAGESAFYVRFEWDGSMGIPAAFHIKNYCQVEFTSRS     143
LOX3_P27481        -------------------------------------------IPGAFYIKNFMQVEFYLKS     19
LOX4_AAF15296      ALGKGKVGSATKLRGQI-SLPTLGASEEAYDVSFDWDADFGIPGAFYIKNFMQNEFYLKS     128
LOX1_P27480        GLGNGKVGKQTFLEKHLPSLPNLGDRQDAFNVYFEWDENFGIPEAFYIKNFMQSEFFLVS     137
LOX2_O24320        GLGNGKVGKQTFLENHLPSLPTLGDRQDAFNISFEWDESFGIPGAFYIKNFMQAEFFLVS     139
                                                               *: ::*  : *:   .

LOX6_ABM88259      IVLDGFEL-GPVRFACESWLHSKHDNPQ-KRIFFPNKSYLPSETPEGVKRLREEELLTLR     233
LOX5_AAG42354      LTLEDVPNQGTIRFVCNSWIYNANIYKKSVRTFFANHSYVPSETPAALVHYREEELKNLR     203
LOX3_P27481        LTLEDIPNHGTIHFICNSWIYNSKVYKS-DRIFFANNTYLPSETPAPLLKYREEELKNVR     78
LOX4_AAF15296      FTLEDIPNYGTIHSICNSWVYNSKKYKS-DRIFFANNTYLPSETPAPLLKYREAELKNVR     187
LOX1_P27480        LTLEDIPNHGTIHFVCNSWVYNAKSYKR-DRIFFANKTYLPNETPASLVKYRKEELENLR     196
LOX2_O24320        LTLEDIPNHGTIHFVCNSWVYNAKNYKK-DRIFFVNKTYVPSETPTPLVKYRKEELENLR     198
                   :.*:..   * ::  *:**::. :      * ** *::*:*.***  : : *: ** .:*

LOX6_ABM88259      GNGQGERQSFERVYDYDVYNDLGDPDENSDLRRPVLGGP-EHPYPRRCRTGRPRTKK--D     290
LOX5_AAG42354      GDGTGERVEHDRIYDYDVYNDLGNPDKAAALARPVLGGS-TLPYPRRGRTGRAKTKKETD     262
LOX3_P27481        GDGSGERKEWDRVYDYDVYNDLGNPDKGAALARPVLGGS-TLPYPRRGRTGRPKTKK--D     135
LOX4_AAF15296      GDGTGKREEWERIYDYDVYNDLGNPDKAAALARPVLGGS-TLPYPRRGRTGRAKTKK--D     244
LOX1_P27480        GDGTGERKEYDRIYDYAVYNDLGNPDKNKNLARTTLGGSSDFPYPRRGRTGRKSTRK--D     254
LOX2_O24320        GDGTGQRKVSDRIYDYDVYNDLGNPDKSADLARPVLGGSSAYPYPRRGRTGRKASKR--D     256
                   *:* *:*   :*:*** ******:**:   * * .***    ***** ****  :::  *

LOX6_ABM88259      PLSEKRSSTVYVPRDECFSEVKQLTFNTKSLASALKALIPALKTVIVD--KNLGFPVFSE     348
LOX5_AAG42354      PNSEKPDDFVYLPRDEAFGHLKSSDFLAYALKSVSQDVLPVLTDAFDGSLLSLEFDNFAE     322
LOX3_P27481        PNSEKPSDFVYLPRDEAFGHLKSSDFLAYGLKSVSQDVLPVLTDAFDGNLLSLEFDNFAE     195
LOX4_AAF15296      PNSEKPDDFVYLPRDEAFGHLKSSDFLAYALKSVSQDVLPVLTDAFDGSLLSLEFDNFAE     304
LOX1_P27480        PKCEIPTSDTYIPRDENFGHLKSGDFLTYAIKSLTQNVLPTFQKAFGF---NNEFDTFED     311
LOX2_O24320        PKSEAPASDTYIPRDENFGHLKSSDFLTYGIKSLAQSVLPQFQSAFGL---NAEFDKFDD     313
                   * .*   . .*:**** *..:*.  * : .: *  : ::* :  .:     .  *  * :

LOX6_ABM88259      IDALFDEGLPLPSRNVKISNLLPRLVSYIKDKGEDILRFNPPATMERDRFFWLRDEEFGR     408
LOX5_AAG42354      VHKLYDGGVTLPTGFLSKYAPIPIVKEIFRSDGEQFLKYPPPKVMQVDKSAWMTDEEFAR     382
LOX3_P27481        VHKLYEGGVTLPTNFLSKYAPIPIVKEIFRSDGEQFLKYPPPKVMQVNKSAWMTDEEFAR     255
LOX4_AAF15296      VHKLYDGGVTLPTGFLSKYAPIPIVKEIFRSDGEQFLKYPPPKVMQVDKSAWMTDEEFAR     364
LOX1_P27480        VRGLFEGGLYLPTDVISKISPIPVLKEILRTDGEQVLKFPPPHVIRVTKSAWMTDEEFGR     371
LOX2_O24320        VRGFFEGGIHLPTDVISNISPLPVIKEIFRTDGEQVLKFPPPHVIQVSKSAWMTDEEFGR     373
                   :  ::: *: **:  :.    :* : . :: .**:.*:: ** .:.  :  *: ****.*

LOX6_ABM88259      QTLAGLNPCCIQLVTEWPLKSKLDPEVYGPAESSITTELVEQEIRGFLTVEEAIKQKKLF     468
LOX5_AAG42354      ETIAGVNPNVIKILKEFPPRSTLDTQAYGDHTSIITKEHLEPKLGG-LTVEQAIENKKLF     441
LOX3_P27481        ETIAGVNPNVIKSLEEFPPRSKLDTQSFGDHTSIITKEHLEINLGG-LTVEQAIQSKKLF     314
LOX4_AAF15296      ETIAGVNPNVIKILKEFPPRSTLDTQAYGDHTSIITKEHLEPKLGG-LTVEQAIENKKLF     423
LOX1_P27480        EMLAGVNPCLIQRLQEFPPKSKLDVTVYGDQTSTMTKEHLEINLGG-LTVEEALHGNRLF     430
LOX2_O24320        EMLAGVNPCLIQRLQEFPPKSKLDASVYGDQTSTITKENLEINLGG-LTVEEALNGNKLF     432
                   : :**:**  *: : *:* :*.**   :*   * :*.* :* :: * ****:*:. ::**
```

```
LOX6_ABM88259      VLDYHDLLLPLVEEVRKLEGTTLYGSRALFFLTEDGTLRPLAIELTRPPYDGKPHW--NR     526
LOX5_AAG42354      ILDHHDYLIPYLRRINS-STTKTYATRTIFFLKDDGTLAPLAIELSKPHSQGDEHGPVSE     500
LOX3_P27481        ILDHHDYLIPYLRRINA-SATKTYATRTIFFLKSDGTLAPLAIELSKPHPQGDEHGPVSE     373
LOX4_AAF15296      ILDHHDYLIPYLRRINS-STTKTYATRTIFFLKDDGTLAPLAIELSKPHPQGDEHGPVSE     482
LOX1_P27480        ILDHHDAFIPYLERINDLPTAKCYATRTILFLKDDNTLKPLAIELSLPNPGGKGAN--SR     488
LOX2_O24320        ILDHHDAFLPYLRKINDLPTAKSYATRTILFLKDDGTLKPLAIELSLPHPRGDEFGAVSR     492
                   :**:** ::* :...:.     :. *.:*:::**..*.** ******: *   *.      ..

LOX6_ABM88259      VLTPTWHATGVWLWRLAKIHIQTHDSGYHQLVSHWLRTHCATEPYIIAANRQMSAMHPIY     586
LOX5_AAG42354      VYVPAYEGVEAYIWLLAKAYVVVNDSCYHQIVSHWLNTHAVVEPFVIATNRQLSVVHPVY     560
LOX3_P27481        VYVPAYEGVEAYIWLLAKAYVVVNDSCYHQLVSHWLNTHAVVEPFVLATNRQLSVVHPVY     433
LOX4_AAF15296      VYVPAYEGVEAYIWLLAKAYVVVNDSCYHQLVSHWLNTHAVVEPFVIATNRQLSVVHPVY     542
LOX1_P27480        VILPADGGAESTIWLLAKAYVVVNDSCYHQLMSHWLNTHAVMEPFVIATNRHLSVLHPIY     548
LOX2_O24320        VILPADQGAESTIWLLAKAYVVVNDSCYHQLMSHWLNTHATIEPFVIATNRHLSVLHPIY     552
                   *  *:  ..   :* *** :: .:** ***::****.**.. **:::*:**::*.:**:*

LOX6_ABM88259      RLLHPHFRYTMEINALARESLINAGGVIEDNFVPGKHSVLLSSIVYDKHWRFDHQALPKD     646
LOX5_AAG42354      KLLFPHYRDTMNINSLARKSLVNADGIIEKTFLWSRYSMEMSSVIYK-DWSLVDQALPND     619
LOX3_P27481        KLLFPHYRDTMNINSLARKSLVNADGIIEKTFLWGRYALELSAVIYK-DWSLHDQALPND     492
LOX4_AAF15296      KLLFPHYRDTMNINSLARKSLVNADGIIEKTFLWGRYALELSAVIYK-DWSLHDQALPND     601
LOX1_P27480        KLLLPHYRDTMNINALARQSLINAGGVIERSFLPGEFAVEMSSAVYK-SWVFTDQALPAD     607
LOX2_O24320        KLLSPHYRDTMNINGLARQSLINAGGIIEQSFLPGPFSVEMSSAVYK-SWVFTDQALPAD     611
                   :** **:* **:**.***:**:**.*:**  .*: . .:: :*: :*.  * : .**** *

LOX6_ABM88259      LIHRGMAVEDPTAPHGLKLTIEDYPYANDGLVLWDALKTWFTEYVDHXYAXSAAVQSDTE     706
LOX5_AAG42354      LIKRGVAVADPSAPHGVKLVIEDYPYASDGLEIWDAIKSWVEEYVAFYYKSDEALQKDPE     679
LOX3_P27481        LVKRGVAVKDPSAPHGVKLVIEDYPYASDGLEIWDAIKSWVVEYVAFYYKSDEVLQQDSE     552
LOX4_AAF15296      LVKRGVAVADPSAPHGVKLVIEDYPYASDGLEIWDAFKSWVVEYVAFYYKSDEVLQQDSE     661
LOX1_P27480        LIKRGMAVEDPSSPYGLRLVVEDYPYAVDGLEIWDTIQTWVKDYVSLYYPTNDAVKKDTE     667
LOX2_O24320        LIKRGMAIEDPSSPHGLRLVIDDYPYAVDGLEIWSAIQSWVKDYVSLYYATDDAIKKDTE     671
                   *::**:*: **::*:*::*.::***** *** :*.::::*. :**   *  . .::.* *

LOX6_ABM88259      LQAWWEEVITLGHADKKDEPWWPELKTKEDLVEIVTTIAWTASGHHAAVNFGQFTFAGYF     766
LOX5_AAG42354      LQAWWKELVQVGHGDLKDKPWWPKMQSRGDLVAVSTTLIWIASALHAAVNFGQYPLGGLI     739
LOX3_P27481        LQAWWKELVQVGHGDLKDKPWWPKMQSRENLVEVSTTLIWIASALHAAVNFGQYPYGGLI     612
LOX4_AAF15296      LQAWWKELVQVGHGDLKDKPWWPKMQSRENLVEVSTTLIWIASALHAAVNFGQYPYGGLI     721
LOX1_P27480        LQAWWKEAVEKGHGDLKDKPWWPKLNTPQDLIHTCSIIIWIASALHAAVNFGQYPYGGFI     727
LOX2_O24320        LQTWWKEAVEKGHGDLKDKPWWPKLNTPQNLIHICSIIIWTASALHAAVNFGQYPYGGYI     731
                   **:**:* :  **.* **:****::::  :*:   : : * **. ********:  .* :

LOX6_ABM88259      PNRPTIVRNNMPIEDPTDPEWELFLQKPEKTMLKSFPSQIQAATVMTVLDILSNHSPDEE     826
LOX5_AAG42354      LNRPTISRRFMPVEGS--AEYAALAKNPEKEFLRTITGKKETLIDLTVIEILSRHASDEI     797
LOX3_P27481        LNRPTISRRFMPEKGS--AEYAALAKNPEKEFLKTITGKKETLIDLTVIEILSRHASDEI     670
LOX4_AAF15296      LNRPTISRRFMPEKGS--AEYAALAKNPEKEFLKTITGKKETLIDLTVIEILSRYASDEI     779
LOX1_P27480        LNRPTITRRLLPEPGT--KEYGELTSNYQKAYLRTITGKVEAIVDLSVIEILSRHASDEV     785
LOX2_O24320        LNRPTLTRRLIPEPGT--KEYDELSSNHQKAYLRTITGKYEAIVDLSVIEILSRHASDEV     789
                    ****: *. :*  .    *:  : .: :*  *::: .: ::   ::*::***.:: **

LOX6_ABM88259      YLGMSVEP-AWEEEPLVNAAFEKFKGKLIELEGLIDARNADTSRKNRNGAGVVPYELLKP     885
LOX5_AAG42354      YLGERDGGDHWTSDAGPLEAFKRFGKKLAEIEEKLVKKNNDETLRNRTGPAKMPYTLLYP     857
LOX3_P27481        YLGERDGGDHWTSDAGPLEAFKRFGKKLAEIEKKLVQKNNDETLRNRTGPAKMPYTLLYP     730
LOX4_AAF15296      YLGERDGGDHWTSDAGPLEAFKRFGKKLAEIEKKLVQKNNDETLRNRTGPAKMPYTLLYP     839
LOX1_P27480        YLGQRDNP-NWTNNIKALQAFKRFGQKLKEIEEKIMGRNKDSSLRNRNGPVKMPYTVLLP     844
LOX2_O24320        YLGQRDNP-NWTDDTKALQAFQKFGNKLKEIENKILGRNNNSSLRNRVGPVKMPYTVLLP     848
                   ***       * .:     **::*  ** *:*  :  :* : : :** *   :** :* *

LOX6_ABM88259      SSD-AGVTGKGVPYSISI      902
LOX5_AAG42354      SSE-EGLTFRGIPNSISI      874
LOX3_P27481        SSE-EGLTFRGI------      741
LOX4_AAF15296      SSE-EGLTFRGIPNSISI      856
LOX1_P27480        TCEDEGLTFRGIPNSISI      862
LOX2_O24320        TSK-EGLTFRGIPNSISI      865
                   :..  *:* :*:
```
